# Serotype distribution of remaining invasive pneumococcal disease after extensive use of ten-valent and 13-valent pneumococcal conjugate vaccines (the PSERENADE project): a global surveillance analysis

**DOI:** 10.1016/S1473-3099(24)00588-7

**Published:** 2024-12-17

**Authors:** Maria Garcia Quesada, Meagan E Peterson, Julia C Bennett, Kyla Hayford, Scott L Zeger, Yangyupei Yang, Marissa K Hetrich, Daniel R Feikin, Adam L Cohen, Anne von Gottberg, Mark van der Linden, Nina M van Sorge, Lucia H de Oliveira, Sara de Miguel, Inci Yildirim, Didrik F Vestrheim, Jennifer R Verani, Emmanuelle Varon, Palle Valentiner-Branth, Georgina Tzanakaki, Nadja Sinkovec Zorko, Lena P Setchanova, Fatima Serhan, Kevin J Scott, J Anthony Scott, Camelia Savulescu, Larisa Savrasova, Rita Reyburn, Kazunori Oishi, J Pekka Nuorti, Daniela Napoli, Jason M Mwenda, Carmen Muñoz-Almagro, Eva Morfeldt, Kimberley McMahon, Allison McGeer, Lucia Mad’arová, Grant A Mackenzie, Maria Eugenia León, Shamez N Ladhani, Karl G Kristinsson, Jana Kozakova, Jackie Kleynhans, Nicola P Klein, James D Kellner, Sanjay Jayasinghe, Pak-Leung Ho, Markus Hilty, Marcella A Harker-Jones, Laura L Hammitt, Marta Grgic-Vitek, Charlotte Gilkison, Ryan Gierke, Neil French, Idrissa Diawara, Stefanie Desmet, Philippe De Wals, Tine Dalby, Ron Dagan, Mary Corcoran, Edoardo Colzani, Grettel Chanto Chacón, Jesús Castilla, Romina Camilli, Michelle Ang, Krow Ampofo, Samanta C G Almeida, Pedro Alarcon, Katherine L O’Brien, Maria Deloria Knoll, The PSERENADE Team

**Affiliations:** Johns Hopkins Bloomberg School of Public Health, Baltimore, MD, USA (M Garcia Quesada MSPH, M E Peterson MD, J C Bennett PhD, K Hayford PhD, Prof S L Zeger PhD, Y Yang MHS, M K Hetrich MHS, Prof L L Hammitt MD, Prof K L O’Brien MD, Prof M Deloria Knoll PhD); WHO, Geneva, Switzerland (D R Feikin MD, A L Cohen MD, F Serhan PhD, Prof K L O’Brien); Centre for Respiratory Diseases and Meningitis, National Institute for Communicable Diseases of the National Health Laboratory Service, Johannesburg, South Africa (Prof A von Gottberg PhD, J Kleynhans PhD); School of Pathology (Prof A von Gottberg) and School of Public Health (J Kleynhans), Faculty of Health Sciences, University of the Witwatersrand, Johannesburg, South Africa; Reference Laboratory for Streptococci, Department of Medical Microbiology, University Hospital RWTH Aachen, Aachen, Germany (M van der Linden PhD); Medical Microbiology and Infection Prevention, Netherlands Reference Laboratory for Bacterial Meningitis, Amsterdam UMC, University of Amsterdam, Amsterdam, Netherlands (Prof N M van Sorge PhD); Pan American Health Organization, WHO, Washington, DC, USA (L H de Oliveira PhD); CIBER de Enfermedades Respiratorias, Madrid, Spain (S de Miguel MD); Epidemiology Department, Dirección General de Salud Pública, Madrid, Spain (S de Miguel); Department of Pediatrics, Yale New Haven Children’s Hospital, New Haven, CT, USA (I Yildirim MD); Department of Public Health, Ministry of Health and Care Services, Oslo, Norway (D F Vestrheim MD); Division of Global Health Protection, Center for Global Health, Centers for Disease Control and Prevention, Nairobi, Kenya (J R Verani MD); Division of Bacterial Diseases, National Center for Immunizations and Respiratory Diseases, US Centers for Disease Control and Prevention, Atlanta, GA, USA (J R Verani, R Gierke MPH); National Reference Centre for Pneumococci, Data Research Department, Intercommunal Hospital of Créteil, Créteil, France (E Varon MD); Infectious Disease Epidemiology and Prevention (P Valentiner-Branth PhD, T Dalby PhD) and Department of Bacteria, Parasites and Fungi (T Dalby), Statens Serum Institut, Copenhagen, Denmark; National Meningitis Reference Laboratory, Department of Public Health Policy, School of Public Health, University of West Attica, Athens, Greece (Prof G Tzanakaki PhD); Communicable Diseases Centre, National Institute of Public Health, Ljubljana, Slovenia (N Sinkovec Zorko MD, M Grgic-Vitek PhD); University Multiprofile Hospital for Active Treatment Saint Ivan Rilski, Clinical Microbiology Laboratory, Sofia, Bulgaria (Prof L P Setchanova PhD); Bacterial Respiratory Infection Service, Scottish Microbiology Reference Laboratory, NHS Greater Glasgow and Clyde, Glasgow, UK (K J Scott PhD); Epidemiology and Demography Department, KEMRI-Wellcome Trust Research Programme, Centre for Geographic Medicine Coast, Kilifi, Kenya (Prof J A Scott FRCP, Prof L L Hammitt); Epidemiology Department, Epiconcept, Paris, France (C Savulescu MD); Institute of Public Health, Riga Stradiņš University, Riga, Latvia (L Savrasova MSc); Murdoch Children’s Research Institute, Parkville, VIC, Australia (R Reyburn MSc); New Vaccines Group, Murdoch Children’s Research Institute, Parkville, VIC, Australia (Prof G A Mackenzie PhD); Toyama Institute of Health, Toyama, Japan (K Oishi MD); Department of Health Security, Finnish Institute for Health and Welfare, Helsinki, Finland (Prof J P Nuorti PhD); Health Sciences Unit, Faculty of Social Sciences, Tampere University, Tampere, Finland (Prof J P Nuorti); Clinical Bacteriology Service, Department of Bacteriology, National Institute for Infectious Diseases (INEI-ANLIS) “Dr Carlos G Malbrán”, Buenos Aires, Argentina (D Napoli BSc); WHO Regional Office for Africa, Brazzaville, Republic of the Congo (Prof J M Mwenda PhD); CIBER Epidemiología y Salud Pública, Madrid, Spain (Prof C Muñoz-Almagro PhD, J Castilla MD); Medicine Department, International University of Catalunya, Barcelona, Spain (Prof C Muñoz-Almagro); Molecular Microbiology Department, Hospital Sant Joan de Déu Research Institute, Barcelona, Spain (Prof C Muñoz-Almagro); Department of Microbiology Public Health Agency of Sweden, Solna, Sweden (E Morfeldt PhD); Centre for Disease Control, Department of Health and Community Services, Darwin, NT, Australia (K McMahon MPH); Toronto Invasive Bacterial Diseases Network and Department of Laboratory Medicine and Pathobiology, University of Toronto, Toronto, ON, Canada (Prof A McGeer MD); National Reference Centre for Pneumococcal and Haemophilus Diseases, Regional Authority of Public Health, Banská Bystrica, Slovakia (L Mad’arová PhD); Department of Paediatrics, University of Melbourne, Parkville, VIC, Australia (Prof G A Mackenzie); Faculty of Infectious and Tropical Diseases, London School of Hygiene & Tropical Medicine, London, UK (Prof G A Mackenzie); Medical Research Council Unit The Gambia at London School of Hygiene & Tropical Medicine, Banjul, The Gambia (Prof G A Mackenzie); Central Laboratory of Public Health, Asunción, Paraguay (M Eugenia León BSc); Immunisation and Countermeasures Division, UK Health Security Agency, London, UK (Prof S N Ladhani PhD); Department of Clinical Microbiology, Landspitali—The National University Hospital, Reykjavik, Iceland (Prof K G Kristinsson PhD); National Institute of Public Health, Prague, Czech Republic (J Kozakova MD); Vaccine Study Center, Kaiser Permanente, Oakland, CA, USA (Prof N P Klein MD); Department of Pediatrics, University of Calgary and Alberta Health Services, Calgary, AB, Canada (Prof J D Kellner MD); National Centre for Immunisation Research and Surveillance and Discipline of Child and Adolescent Health, Children’s Hospital Westmead Clinical School, Faculty of Medicine and Health, University of Sydney, Westmead, NSW, Australia (S Jayasinghe PhD); Department of Microbiology and Carol Yu Centre for Infection, Queen Mary Hospital, The University of Hong Kong, Hong Kong Special Administrative Region, China (P-L Ho MD); Swiss National Reference Centre for Invasive Pneumococci, Institute for Infectious Diseases, University of Bern, Bern, Switzerland (M Hilty PhD); Arctic Investigations Program, Division of Preparedness and Emerging Infections, National Center for Emerging and Zoonotic Infectious Diseases, US Centers for Disease Control and Prevention, Anchorage, AK, USA (M A Harker-Jones BS); Epidemiology Team, Institute of Environmental Science and Research, Porirua, New Zealand (C Gilkison MPH); Institute of Infection, Veterinary and Ecological Sciences, University of Liverpool, Liverpool, UK (Prof N French PhD); Malawi Liverpool Wellcome Programme, Blantyre, Malawi (Prof N French); Infectious Diseases Research Unit, Mohammed VI Center for Research & Innovation (CM6RI), Rabat, Morocco (Prof I Diawara PhD); Mohammed VI University of Sciences and Health, Mohammed VI Higher Institute of Biosciences and Biotechnologies (UM6SS), Casablanca, Morocco (Prof I Diawara); Department of Microbiology, Immunology and Transplantation, KU Leuven, Leuven, Belgium (S Desmet PhD); National Reference Centre for Streptococcus Pneumoniae, University Hospitals Leuven, Leuven, Belgium (S Desmet); Department of Social and Preventive Medicine, Laval University, Québec, QC, Canada (Prof P De Wals PhD); The Shraga Segal Department of Microbiology, Immunology and Genetics, Faculty of Health Sciences, Ben-Gurion University of the Negev, Beer-Sheva, Israel (Prof R Dagan MD); Irish Meningitis and Sepsis Reference Laboratory, Children’s Health Ireland at Temple Street, Dublin, Ireland (M Corcoran PhD); Royal College of Surgeons in Ireland, Dublin, Ireland (M Corcoran); European Centre for Disease Prevention and Control, Solna, Sweden (E Colzani PhD); Costa Rican Institute for Research and Teaching in Nutrition and Health, Tres Ríos, Costa Rica (G Chanto Chacón MS); Public Health Institute of Navarre, Pamplona, Spain (J Castilla); Navarre Institute for Health Research, Pamplona, Spain (J Castilla); Department of Infectious Diseases, Italian National Institute of Health, Rome, Italy (R Camilli PhD); National Public Health Laboratory, National Centre for Infectious Diseases, Singapore (M Ang PhD); Division of Pediatric Infectious Diseases, Department of Pediatrics, University of Utah Health Sciences Center, Salt Lake City, UT, USA (Prof K Ampofo MBChB); National Laboratory for Meningitis and Pneumococcal Infections, Center of Bacteriology, Institute Adolfo Lutz, São Paulo, Brazil (S C G Almeida PhD); Instituto de Salud Pública de Chile, Santiago, Chile (P Alarcon MPH)

## Abstract

**Background:**

Widespread use of pneumococcal conjugate vaccines (PCVs) has reduced vaccine-type invasive pneumococcal disease (IPD). We describe the serotype distribution of IPD after extensive use of ten-valent PCV (PCV10; Synflorix, GSK) and 13-valent PCV (PCV13; Prevenar 13, Pfizer) globally.

**Methods:**

IPD data were obtained from surveillance sites participating in the WHO-commissioned Pneumococcal Serotype Replacement and Distribution Estimation (PSERENADE) project that exclusively used PCV10 or PCV13 (hereafter PCV10 and PCV13 sites, respectively) in their national immunisation programmes and had primary series uptake of at least 70%. Serotype distribution was estimated for IPD cases occurring 5 years or more after PCV10 or PCV13 introduction (ie, the mature period when the serotype distribution had stabilised) using multinomial Dirichlet regression, stratified by PCV product and age group (<5 years, 5–17 years, 18–49 years, and ≥50 years).

**Findings:**

The analysis included cases occurring primarily between 2015 and 2018 from 42 PCV13 sites (63 362 cases) and 12 PCV10 sites (6806 cases) in 41 countries. Sites were mostly high income (36 [67%] of 54) and used three-dose or four-dose booster schedules (44 [81%]). At PCV10 sites, PCV10 serotypes caused 10·0% (95% CI 6·3–12·9) of IPD cases in children younger than 5 years and 15·5% (13·4–19·3) of cases in adults aged 50 years or older, while PCV13 serotypes caused 52·1% (49·2–65·4) and 45·6% (40·0–50·0), respectively. At PCV13 sites, PCV13 serotypes caused 26·4% (21·3–30·0) of IPD cases in children younger than 5 years and 29·5% (27·5–33·0) of cases in adults aged 50 years or older. The leading serotype at PCV10 sites was 19A in children younger than 5 years (30·6% [95% CI 18·2–43·1]) and adults aged 50 years or older (14·8% [11·9–17·8]). Serotype 3 was a top-ranked serotype, causing about 9% of cases in children younger than 5 years and 14% in adults aged 50 years or older at both PCV10 and PCV13 sites. Across all age and PCV10 or PCV13 strata, the proportion of IPD targeted by higher-valency PCVs beyond PCV13 was 4·1–9·7% for PCV15, 13·5–36·0% for PCV20, 29·9–53·8% for PCV21, 15·6–42·0% for PCV24, and 31·5–50·1% for PCV25. All top-ten ranked non-PCV13 serotypes are included in at least one higher-valency PCV.

**Interpretation:**

The proportion of IPD due to serotypes included in PCVs in use was low in mature PCV10 and PCV13 settings. Serotype distribution differed between PCV10 and PCV13 sites and age groups. Higher-valency PCVs target most remaining IPD and are expected to extend impact.

**Funding:**

Bill & Melinda Gates Foundation as part of the WHO Pneumococcal Vaccines Technical Coordination Project.

## Introduction

*Streptococcus pneumoniae* is a major cause of morbidity and mortality among young children and older adults, with an estimated 829 000 deaths attributed to pneumococcus globally in 2019, of which 225 000 were among children younger than 5 years.^1^ Before the introduction of pneumococcal conjugate vaccines (PCVs), more than 70% of invasive pneumococcal disease (IPD) cases were estimated to have been caused by serotypes targeted by ten-valent and 13-valent PCVs.^2^ PCVs first became available in 2000 with a seven-valent vaccine (PCV7; Prevenar, Pfizer, New York, NY, USA); in 2007, WHO recommended the inclusion of PCVs in national childhood immunisation programmes globally, which as of 2024 has been implemented by 170 countries.^3,4^ As a result, vaccine-type disease has been greatly reduced through direct protection of vaccinated children and indirect protection of unvaccinated individuals resulting from decreased carriage and circulation of these serotypes in the general population.^5,6^ Some of these gains have been offset by replacement disease, caused by serotypes not covered by the vaccines (non-vaccine types).^6^

A ten-valent PCV (PCV10; Synflorix, GSK, Coleford, UK) and a 13-valent PCV (PCV13; Prevenar 13, Pfizer) have been available since 2010 and are widely used. Questions remain regarding whether one prevents more disease than the other and the potential impact of new higher-valency vaccines on remaining disease. Several new PCVs have been recently licensed for use in infants (appendix 2 p 10): another ten-valent PCV (Pneumosil, Serum Institute of India, Pune, India), which received WHO prequalification in 2019^7^ and differs slightly from Synflorix in the serotypes covered; a 15-valent PCV (PCV15; VAXNEUVANCE, Merck, Darmstadt, Germany), licensed for children in 2022, which includes PCV13 serotypes and two additional serotypes;^8,9^ and a 20-valent PCV (PCV20; Prevenar 20, Pfizer), licensed for children in 2023–24, which also includes PCV13 serotypes and seven additional serotypes.^10,11^ PCV15 and PCV20 are also licensed for adults, as is a 21-valent PCV (PCV21; V116 and CAPVAXIVE, Merck), which excludes PCV10 serotypes except serotype 7F.^12^ A 23-valent pneumococcal polysaccharide vaccine (PPV23; Pneumovax23, Merck) is licensed for individuals aged 2 years or older and is recommended in many countries for older adults, but is not an infant vaccine. Other higher-valency PCVs in the pipeline include multiple 24-valent PCVs (GSK, Vaxcyte, and Merck),^13–15^ which include all PCV13 types and target the same serotypes as PPV23 plus 6A, and a 25-valent PCV (IVT PCV-25, Inventprise, Redmond, WA, USA),^16,17^ which includes all PCV13 types except serotype 6A (appendix 2 p 10).

To evaluate the effect of long-term routine use of PCV10 and PCV13 on IPD incidence, and to estimate the remaining IPD due to serotypes covered by higher-valency vaccines among all ages, WHO commissioned the Pneumococcal Serotype Replacement and Distribution Estimation (PSERENADE) project, a collaboration of more than 50 institutions.^18^ In this study, we aimed to estimate the global serotype distribution of IPD in settings where PCV10 or PCV13 have been used extensively, particularly for children younger than 5 years of age and older adults. We present the proportion of IPD due to serotypes covered by the PCV product used and assess the potential impact of the newer, higher-valency PCVs.

## Methods

### Site identification

Site identification and data collection methods are described in a previous publication.^18^ Briefly, various methods were used to identify sites conducting serotype-specific IPD surveillance where PCV10 (Synflorix unless otherwise specified, as Pneumosil was not in use at the time of project initiation) or PCV13 was universally recommended for infants for at least 1 year by 2018. Institutions that collected IPD data, including research groups and national laboratory testing centres, were invited to participate. Institutions in agreement shared their data in 2018 or 2019. IPD was defined as *S pneumoniae* isolated by culture from any normally sterile fluid or using *lytA*-based PCR or antigen-based tests in cerebrospinal or pleural fluid. This activity was reviewed by the Johns Hopkins Institutional Review Board and US Centers for Disease Control and Prevention, deemed research not involving human subjects and exempt from institutional review board oversight, and conducted consistent with applicable federal law and US Centers for Disease Control and Prevention policy.

The datasets provided by the institutions conducting the surveillance underwent extensive quality checks by PSERENADE analysts to identify potential biases that could affect the serotype distribution, including selective serotyping of cases or incomplete serotyping methods that might result in a non-representative serotype distribution of all IPD. These evaluations and resulting decisions on whether to include the data were discussed with site investigators.^18^ Site characteristics and serotyping methods are described in appendix 2 (p 11).

### Data eligibility and defining the mature period

Site data eligible for the first stage of defining the mature period had serotyped at least 50% of isolates and at least 12 months of continuous surveillance for 4 years or more after PCV10 or PCV13 introduction (including the year of introduction). The mature period was defined as when the serotype distribution stabilised. To determine the number of years of exclusive and continuous PCV10 or PCV13 use required to reach the mature period, the data from each site were assessed by age group; the evidence across all sites was evaluated to determine thresholds specific to age group. For children, the mature PCV10 or PCV13 period was determined to be reached after 7 years (including the year of introduction), unless there was a PCV10 or PCV13 catch-up programme, which reduced the time to 6 years, or unless another PCV product (eg, PCV7) was used previously for 3 years or more, which reduced it to 5 years. For adults, the mature PCV10 or PCV13 period was reached after 7 years regardless of other previous PCV use or catch-up activities in children. Data from years in the mature period from sites with at least 70% PCV10 or PCV13 uptake were included in the primary analyses. Eligibility steps are described in more detail in a previous pneumococcal meningitis analysis.^19^ A sensitivity analysis of the mature period was conducted by including only data collected at least 7 years after PCV10 or PCV13 introduction, regardless of previous PCV use or catch-up.

### Defining serotype categories

Cases were grouped into the following serotype categories: PCV7 (4, 6B, 9V, 14, 18C, 19F, and 23F); PCV10 (PCV7 plus 1, 5, and 7F); PCV13 (PCV10 plus 3, 6A, and 19A); PCV15 (PCV13 plus 22F and 33F);^8^ PCV20 (PCV15 plus 8, 10A, 11A, 12F, and 15BC);^10^ PCV24 (PCV20 plus 2, 9N, 17F, and 20);^13–15^ PCV25 (PCV20 minus 6A and 11A, but plus 2, 6C, 9N, 15A, 16F, 24F, and 35B);^17^ PPV23 (PCV24 minus 6A); PCV21 (3, 6A, 7F, 8, 9N, 10A, 11A, 12F, 15A, 15BC, 16F, 17F, 19A, 20, 22F, 23A, 23B, 24F, 31, 33F, and 35B);^20^ and a single category of PCV10 or PCV21 (any of the 30 serotypes in PCV10 or PCV21, which includes all serotypes in PCV13). We were unable to estimate the serotype coverage of Pneumosil because PCV10 (Synflorix) and PCV13 target serotypes 4 and 18C, which are not covered by Pneumosil.^21^ Because PCV21 is targeted at adults, we could estimate PCV21 serotype coverage as we assumed that it will be used in conjunction with another PCV among infants that targets the nine PCV10 serotypes not covered by PCV21.^12^ Serotypes 15B and 15C were grouped together as 15BC because most sites do not distinguish between the two; the serotypes can switch due to a slipped strand mispairing of a tandem thymine–adenine repeat.^22^ Non-vaccine-type serotypes were defined as serotypes not in the indicated vaccine. Cases without a specific serotype identified were rare and were grouped into four categories defined in a previous publication as not serotyped; untypeable; typed, serotype not identified; and serogrouped only.^19^

### Analytical model

The predicted probability of IPD in the mature PCV10 or PCV13 period due to serotype categories and specific serotypes was estimated using multinomial Dirichlet regression, as described elsewhere, which produced a weighted mean of serotype distributions across sites.^19,23^ The model considered the sample size of each site and the heterogeneity in the serotype distribution between sites to assign site-specific weights; the greater the heterogeneity, the more similarly sites are weighted, and the lower the heterogeneity, the more they are weighted proportional to sample size. The serotype distribution for older adults included adults aged 50 years or older to increase sample size, because no differences were observed between those aged 50–64 years and 65 years or older. The model included a covariate for PCV product to estimate separate distributions for PCV10 and PCV13 sites, and a covariate for age to generate distributions for smaller age groups among children (ages 0–23 months and 24–59 months) and adults (ages 50–64 years, 65–74 years, 75–84 years, and ≥85 years) at PCV13 sites; data from PCV10 sites were insufficient to estimate for finer age strata. Data were also insufficient to estimate modelled distributions by UN region, so regional differences were analysed descriptively.

We estimated proportions of IPD caused by 41 serotypes, including those in PCV24, 6C due to its possible cross-protection from 6A,^24^ and 16 high-frequency serotypes not included in PCV24. We could not estimate proportions for all observed serotypes due to statistical limitations caused when sites have many serotypes with zero counts. To identify high-frequency serotypes not included in PCV24, data were first pooled across sites to estimate the rank of non-PCV24 serotypes by product and age group, and then the top-ten ranked serotypes in any age group (<5 years and ≥50 years) and product (PCV10 and PCV13) stratum were selected.

To understand the relative frequency of PCV13-type and non-PCV13-type serotypes between PCV10 and PCV13 sites, which had very different proportions of PCV13-type IPD, we estimated the serotype distribution of PCV13-type (plus serotype 6C) and non-PCV13-type serotypes separately, using a denominator restricted to those serotypes (eg, we estimated the serotype distribution of non-PCV13 serotypes among non-PCV13 serotypes). To assess the influence of very small and very large sites on the modelled results, sensitivity analyses were conducted excluding sites with fewer than 20 cases or very large sites (eg, England and Wales) individually.

Analyses were performed using R version 4.3.3, and for modelling the VGAM package was used (appendix 2 p 2).^23^

### Role of the funding source

The invitation to participate was sent on behalf of WHO. The funders reviewed and commented on the manuscript but had no role in study design, data collection, data analysis, data interpretation, writing of the manuscript, or the decision to submit for publication.

## Results

**Figure 1:**
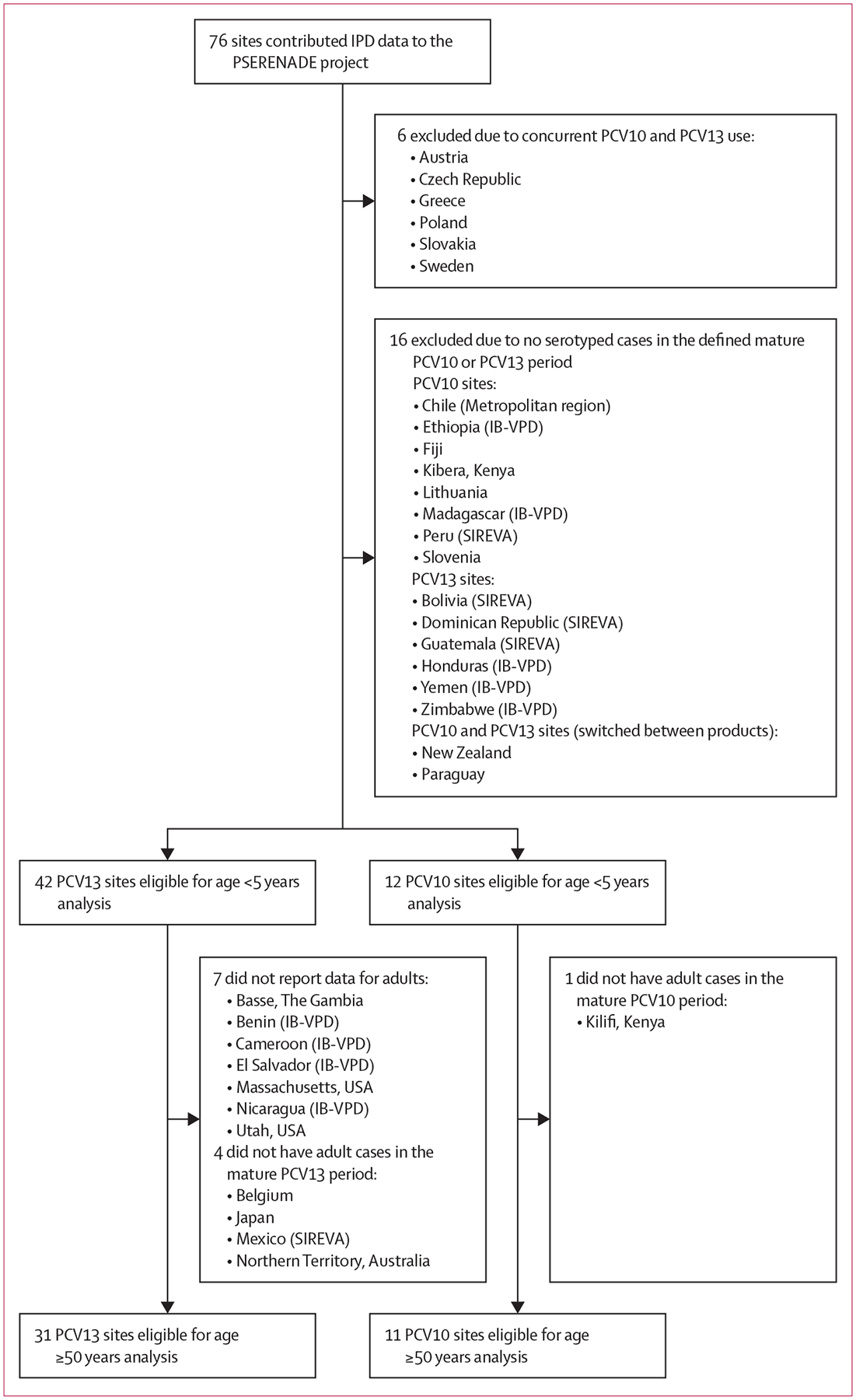
Sites that contributed IPD data to the PSERENADE project and reasons for exclusion from analysis IB-VPD=WHO Global Invasive Bacterial Vaccine-Preventable Diseases Laboratory Network. IPD=invasive pneumococcal disease. PCV=pneumococcal conjugate vaccine. PCV10=ten-valent PCV (Synflorix, GSK). PCV13=13-valent PCV (Prevenar 13, Pfizer). SIREVA=Pan American Health Organization Regional System for Vaccines.

Of 76 sites that contributed IPD data to the PSERENADE project, 54 (71%) were eligible for analysis (63 362 cases from 42 PCV13 sites and 6806 cases from 12 PCV10 sites; figure 1; appendix 2 p 11). Six sites were excluded due to concurrent PCV10 and PCV13 use, and 16 were excluded because they had no serotyped IPD cases in the defined mature PCV10 or PCV13 period. 28 (52%) eligible sites were from Europe or North America. Of the 18 (33%) sites from low-income and middle-income countries, only five had more than 20 eligible cases for children and seven had more than 20 eligible cases for adults. All eligible sites had eligible data for the analysis of children younger than 5 years (7318 cases from PCV13 sites and 1109 cases from PCV10 sites; table). For adults aged 50 years or older, 31 PCV13 sites (43 774 cases) and 11 PCV10 sites (3978 cases) had data eligible for analysis; seven did not report data for adults and five did not have data in the mature PCV10 or PCV13 period as defined for adults (figure 1). Ten sites did not use a booster dose schedule (3 + 0), of which only five had eligible data for adults, and most of these had fewer than 20 eligible cases (eight of ten for children and three of five for adults; table). Specific years of included data varied by site and age group, but primarily represented the years 2015–18. Additional site details and characteristics are described in appendix 2 (p 11) and elsewhere.^18^

**Figure 2:**
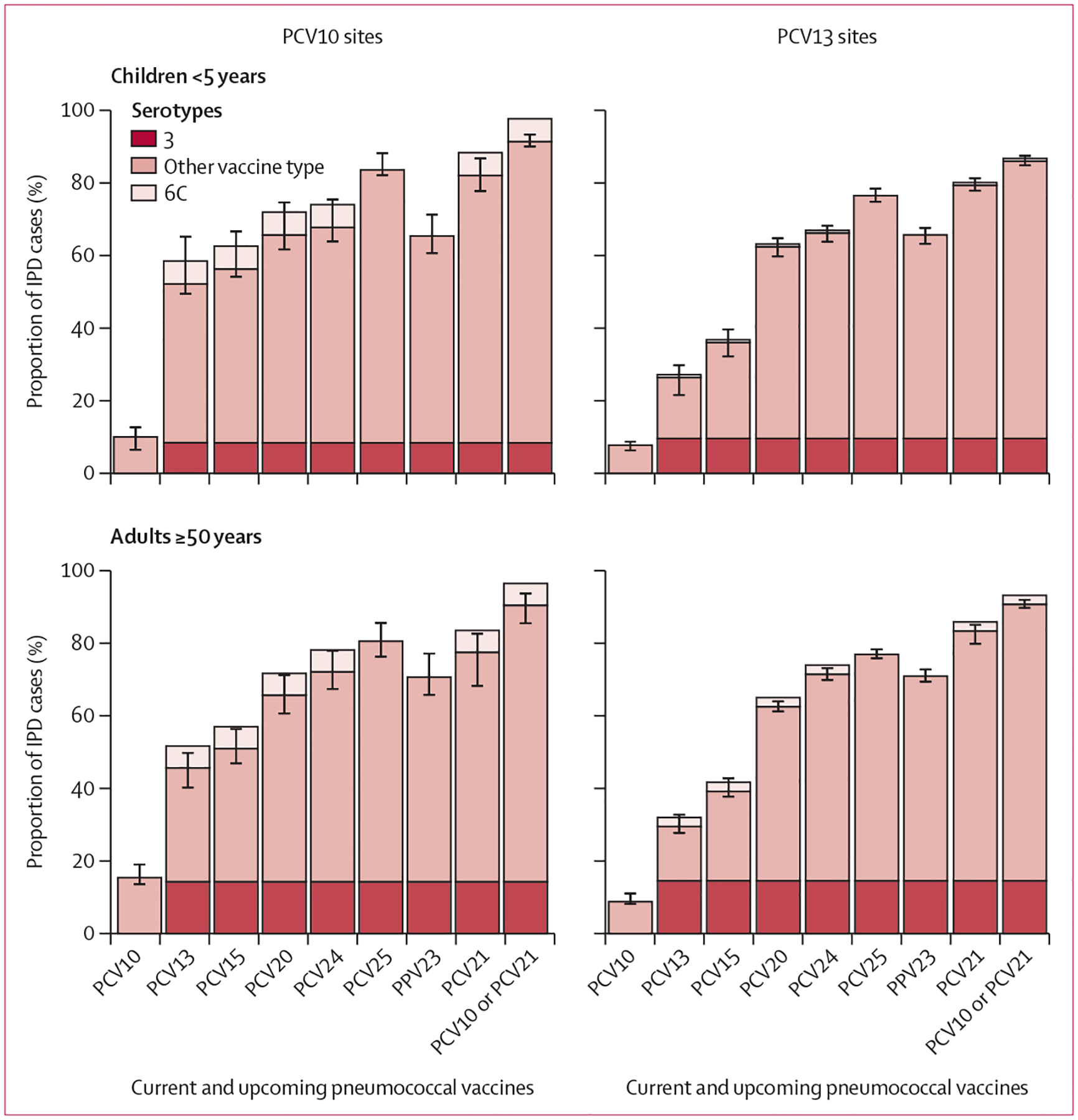
Proportion of IPD cases in the mature PCV10 or PCV13 period due to serotypes included in current and upcoming products The tops of the bars indicate weighted means, with error bars representing 95% CIs. Serotype 3 is illustrated separately from other vaccine-type serotypes for products that include serotype 3 due to the uncertain effectiveness of current products against serotype 3. Serotype 6C is illustrated above the bars for products containing serotype 6A but not 6C as evidence suggests possible cross-protection against serotype 6C from 6A;^24^ serotype 6C was not included in estimation of the weighted means and 95% CIs (except for PCV25, the only vaccine in which it is included). Serotypes covered by each vaccine are shown in appendix 2 (p 10); higher-valency vaccines include most serotypes of lower-valency vaccines. IPD=invasive pneumococcal disease. PCV=pneumococcal conjugate vaccine. PCV10=ten-valent PCV (Synflorix, GSK). PCV13=13-valent PCV (Prevenar 13, Pfizer). PCV15=15-valent PCV (VAXNEUVANCE, Merck). PCV20=20-valent PCV (Prevenar 20, Pfizer). PCV21=21-valent PCV (V116 and CAPVAXIVE, Merck). PCV24=24-valent PCVs (GSK, Vaxcyte, and Merck). PCV25=25-valent PCV (IVT PCV-25, Inventprise). PPV23=23-valent pneumococcal polysaccharide vaccine.

**Figure 3:**
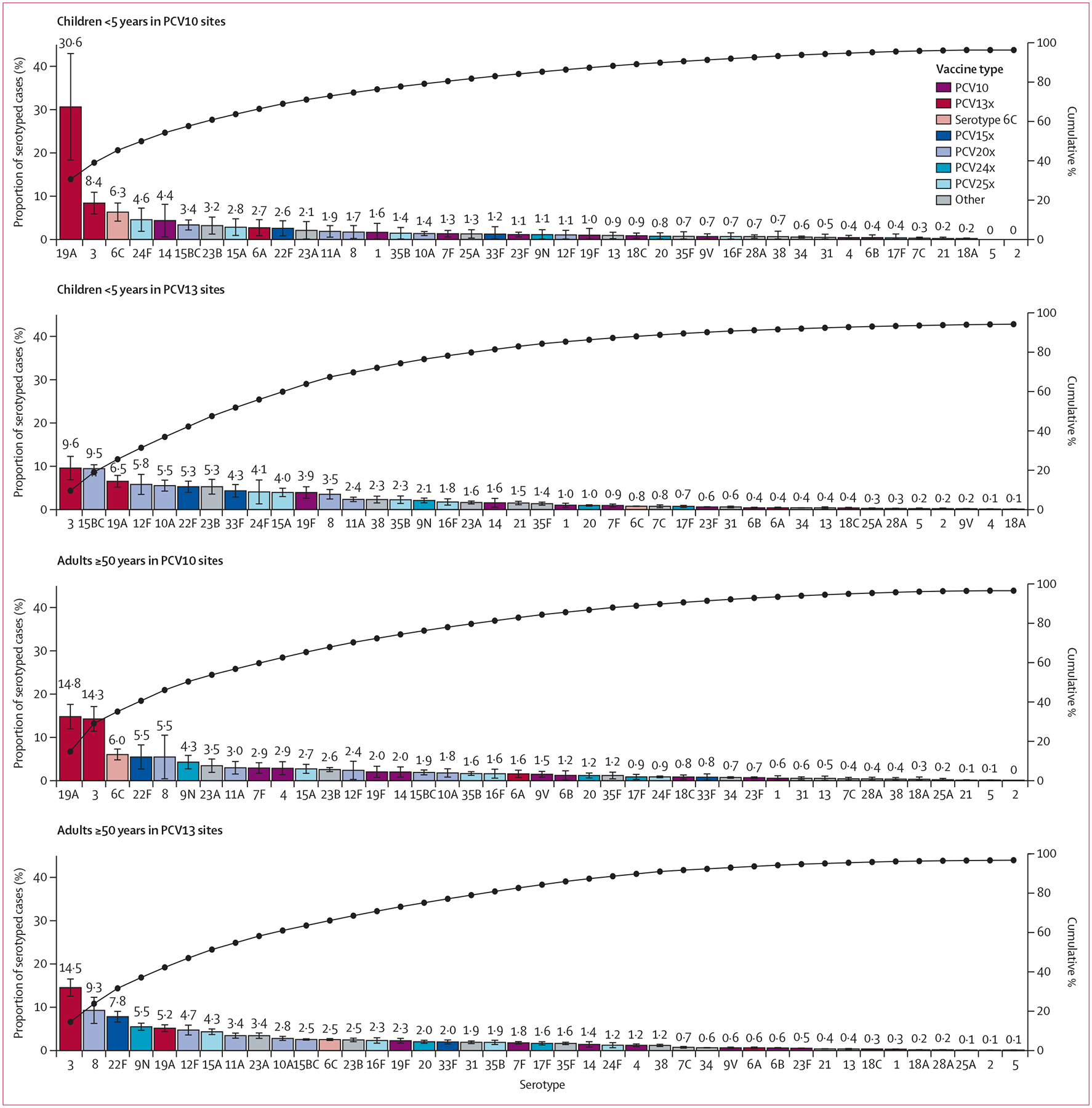
Serotype-specific distribution of IPD cases in the mature PCV10 or PCV13 period The tops of the bars indicate weighted means, with error bars representing 95% CIs. Serotypes are coloured by the lowest-valency PCV product in which they are included. The “x” in the key represents the extra serotypes included in that product relative to the next lower-valency product (eg, PCV13x includes serotypes 3, 6A, and 19A, which are not in PCV10). The only exception is PCV25x—not all serotypes in PCV24 are in PCV25, so PCV25x represents multiple serotypes that are in PCV25 but not PCV24 (see appendix 2 p 10 for differences in coverage). Serotype 6C is shown separately because, although it is not included in most products (except PCV25), it is potentially covered through cross-protection with PCV13-type serotype 6A.^24^ PCV21, which excludes nine PCV10 types, is not shown here (appendix 2 p 26), but other types covered by PCV21 include 23A, 23B, and 31. Serotypes 15B and 15C were grouped as 15BC as described in Methods. IPD=invasive pneumococcal disease. PCV=pneumococcal conjugate vaccine. PCV10=ten-valent PCV (Synflorix, GSK). PCV13=13-valent PCV (Prevenar 13, Pfizer). PCV15=15-valent PCV (VAXNEUVANCE, Merck). PCV20=20-valent PCV (Prevenar 20, Pfizer). PCV21=21-valent PCV (V116 and CAPVAXIVE, Merck). PCV24=24-valent PCVs (GSK, Vaxcyte, and Merck). PCV25=25-valent PCV (IVT PCV-25, Inventprise). PPV23=23-valent pneumococcal polysaccharide vaccine.

At PCV10 sites, PCV10 serotypes caused 10·0% (95% CI 6·3–12·9) of IPD cases among children younger than 5 years and 15·4% (13·4–19·3) among adults aged 50 years and older (figure 2, appendix 2 p 14). PCV10-type IPD was primarily due to serotype 14 in children (4·4%, ranked fifth among all serotypes in children in PCV10 sites) and serotypes 7F and 4 in adults (2·9% each, ranked ninth and tenth; figure 3, appendix 2 p 15). Serotype 1, which is known to cause outbreaks, was rarely seen at any age or product stratum (0·3–1·6%). For PCV13 sites, 26·4% (21·3–30·0) of IPD cases were caused by PCV13 serotypes among children and 29·5% (27·5–33·0) among adults (figure 2). The main PCV13 serotype causing IPD, and the top serotype overall, was serotype 3 for both children (9·6% [6·8–12·4]) and adults (14·5% [12·4–16·7]), followed by 19A (6·5% [ranked third] for children and 5·2% [ranked fifth] for adults; figure 3). The proportion of PCV13-type IPD was greater at PCV10 sites than at PCV13 sites in both children (52·1% *vs* 26·4%) and adults (45·6% *vs* 29·5%; figure 2), largely due to serotype 19A, which caused 30·6% (18·2–43·1) of IPD in children at PCV10 sites and 14·8% (11·9–17·8) in adults (figure 3). The contribution of serotype 6C, potentially covered by serotype 6A in PCV13 but not PCV10, was also higher at PCV10 sites than PCV13 sites (6·3% *vs* 0·8% in children and 6·0% *vs* 2·5% in adults). Similar to PCV13 sites, serotype 3 caused 8·4% (5·8–11·0) of IPD in children and 14·3% (11·3–17·3) in adults at PCV10 sites.

In aggregate, IPD serotype coverage across both age groups and PCV-use groups was 36·0–56·3% for PCV15, 62·4–65·6% for PCV20, 66·2–72·1% for PCV24, 76·5–83·6% for PCV25, and 65·4–70·9% for PPV23 (figure 2, appendix 2 p 14). The five additional serotypes in PCV15 caused 46·3% of IPD beyond PCV10 among children and 35·5% among adults at PCV10 sites, and 9·6% beyond PCV13 among children and adults at PCV13 sites. The additional serotypes in PCV20 beyond PCV15 caused 9·3–14·7% of IPD in PCV10 sites and 23·4–26·4% in PCV13 sites. These proportions were more similar between PCV10 and PCV13 sites when PCV13 serotypes and serotype 6C were excluded from the denominator (appendix 2 p 24). Serotypes included in PCV21, which only include one PCV10 type (7F), caused 77·5–83·3% of IPD across all age and product strata; serotype coverage of PCV21 and PCV10 (or PCV13) combined was 86·0–91·4% (appendix 2 p 14). Across age groups, 74·5–80·7% of IPD cases at PCV10 sites were caused by the serotypes in PCV21 beyond PCV10 types (ie, all but 7F, which is included in PCV10), and 60·6–61·7% of IPD cases at PCV13 sites were caused by the serotypes in PCV21 beyond PCV13 types (ie, all but 3, 6A, 7F, and 19A, which are included in PCV13; appendix 2 p 15).

**Figure 4:**
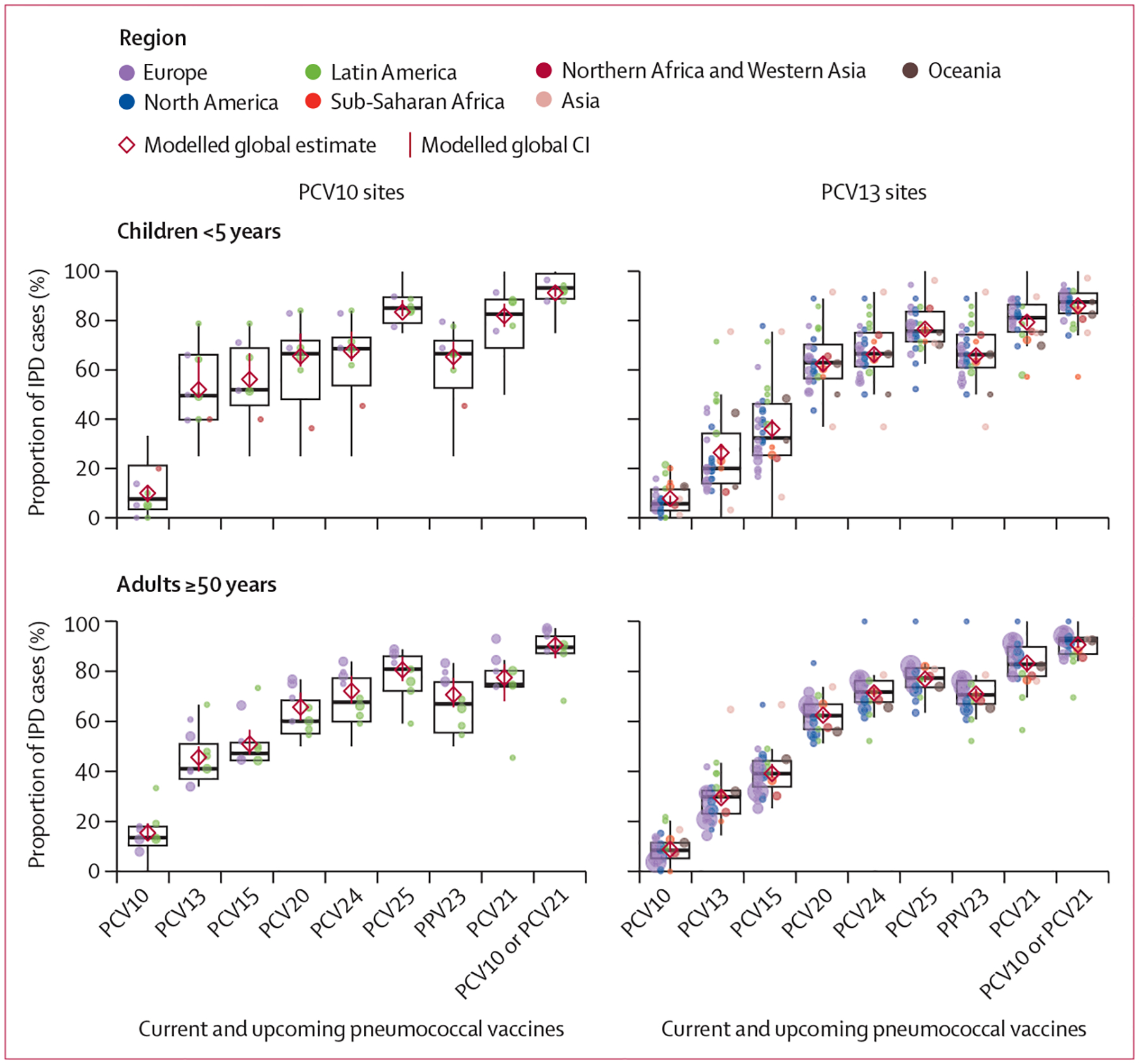
Site-specific proportions of IPD cases in the mature PCV10 or PCV13 period due to serotypes included in current and upcoming products Each site is represented by a dot, coloured by region and sized proportionally to the number of cases contributed by that site. Box plots indicate medians with IQRs for the site-specific percentages. The modelled weighted means shown in figure 2 are shown here by red diamonds. Sites with fewer than five cases are not shown but were included in the modelled and box plot estimates. Data from Singapore are not shown for confidentiality but contributed to the PCV13 modelled results. IPD=invasive pneumococcal disease. PCV=pneumococcal conjugate vaccine. PCV10=ten-valent PCV (Synflorix, GSK). PCV13=13-valent PCV (Prevenar 13, Pfizer). PCV15=15-valent PCV (VAXNEUVANCE, Merck). PCV20=20-valent PCV (Prevenar 20, Pfizer). PCV21=21-valent PCV (V116 and CAPVAXIVE, Merck). PCV24=24-valent PCVs (GSK, Vaxcyte, and Merck). PCV25=25-valent PCV (IVT PCV-25, Inventprise). PPV23=23-valent pneumococcal polysaccharide vaccine.

At least 13 of the most common serotypes for each age group and PCV setting are included in at least one higher-valency PCV (figure 4, appendix 2 p 26). Among children, serotype 24F was the most common non-PCV13 type at PCV10 sites (4·6% [95% CI 1·8–7·4]) and 15BC at PCV13 sites (9·5% [8·4–10·5]). Among adults, serotypes 8, 9N, and 22F were the most common non-PCV13 serotypes, together causing 15·3% of cases at PCV10 sites and 22·6% at PCV13 sites.

At PCV13 sites, children aged 0–23 months had a lower proportion of PCV13-type IPD (22·5% [95% CI 18·3–26·0]) than children aged 24–59 months (33·4% [25·5–37·1]; appendix 2 p 16), due primarily to serotypes 1, 3, and 19A (appendix 2 pp 17, 29). Serotypes in PCV15 (33·1% *vs* 40·7%), PCV20 (61·2% *vs* 64·9%), PCV24 (65·9% *vs* 67·7%), and PCV25 (75·9% *vs* 77·8%) were more similar between age groups (appendix 2 p 16). Non-PCV13 serotypes 10A and 33F were more common among younger children, whereas 12F and 23B were more common among older children. Data at PCV10 sites were insufficient for further age stratification (appendix 2 p 27).

When stratifying adults in PCV13 sites by finer age groups, serotypes covered by PCV10 (range across age groups 7·5% to 9·7%), PCV13 (26·9% to 27·8%), PCV15 (37·0% to 37·9%), and PCV21 (83·8% to 85·4%) caused generally similar proportions of disease among groups, whereas serotype coverage appeared to decline with age for PCV20 (65·8% for age 50–64 years to 58·1% for age ≥85 years), PCV24 (76·2% to 66·4%), PCV25 (79·5% to 75·3%), and PPV23 (75·8% to 65·8%; appendix 2 p 16). Declines by age in serotype coverage by higher-valency vaccines were largely due to serotypes 8 (from 12·6% of cases in adults aged 50–64 years to 7·3% for age ≥85 years), 9N (6·7% to 5·3%), and 12F (6·4% to 3·7%), but CIs were wide. Non-PPV23 serotypes generally increased with age (appendix 2 pp 17, 29).

Data were insufficient to further stratify serotype distributions already stratified by product and age by UN region. However, heterogeneity in the proportion of IPD covered by the different products across sites within the same region was generally wide, in part due to small sample sizes, and overlapped across sites from other regions (figure 4, appendix 2 p 18). No clear regional patterns were apparent in the proportion of vaccine-type IPD (or other PCV valency) across sites according to their region, except that a greater number of PCV13 sites in Latin America had more PCV13-type IPD than North American or European sites for children younger than 5 years. At PCV10 sites, the proportion of IPD among adults due to PCV20, PCV24, and PCV25 types and PPV23 types was lower in Latin American than European sites (figure 4).

Heterogeneity across sites in serotype-specific ranking and proportions was also observed, both within and across regions (appendix 2 p 30). Heterogeneity was unlikely to be due to differences in the age distributions of IPD cases, which were generally similar across sites (appendix 2 p 27).

When estimating PCV serotype coverage using median instead of the modelled weighted mean, estimates were generally within 5% (figure 4). Sensitivity analyses that excluded small sites (<20 cases), excluded large sites (eg, England and Wales) individually, or restricted the mature period to 7 years or more after introduction of PCV10 or PCV13 did not meaningfully influence results (data not shown).

## Discussion

This global analysis estimated the serotype distribution of IPD in children and adults in mature PCV10 or PCV13 settings after approximately 7 years in national childhood immunisation programmes with high (≥70%) uptake. Serotypes covered by PCV10 and PCV13 caused a minority of remaining IPD (10–15% of cases were due to PCV10 serotypes in PCV10 sites and 26–30% were due to PCV13 serotypes in PCV13 sites) but were not eliminated. The proportion of IPD cases caused by PCV13 serotypes was higher at PCV10 sites than PCV13 sites, predominantly due to serotypes 19A, 3, and 6C, suggesting that use of a PCV that protects against those serotypes can further reduce disease at sites currently using PCV10. However, serotype 3 was the most common serotype at PCV13 sites, suggesting that a different technology to the one used in PCV13 is needed to protect against serotype 3.^21^ New paediatric PCVs with 20–25 serotypes cover much remaining IPD (eg, for children aged <5 years, up to 74% of all IPD beyond PCV10 types at PCV10 sites and 50% beyond PCV13 types at PCV13 sites).

Serotypes covered by PCV13 represented a larger proportion of all IPD cases than of meningitis cases only, which was assessed in a previous analysis, attributed mostly to serotypes 19A and 3.^19^ Three multicountry IPD studies estimated the serotype distribution of IPD cases occurring after PCV10 or PCV13 introduction in the general population, excluding cases that occurred early after introduction. Two of these studies assessed cases in high-income countries occurring at least 3 years after PCV10 or PCV13 introduction, one in children younger than 5 years and one in adults primarily aged 65 years or older,^25,26^ and one study assessed cases occurring in European countries in both of those age groups annually from 2011, when most countries introduced PCV10 or PCV13, up to 2018.^27^ Compared with our study, the studies in high-income countries estimated higher proportions of IPD cases due to PCV10 or PCV13 serotypes at sites exclusively using PCV10 (3–8% higher) and similar proportions at sites exclusively using PCV13 (1–2% lower) for both children and older adults. By contrast, the European study estimated lower proportions of IPD cases due to PCV10 or PCV13 serotypes at both PCV10 and PCV13 sites than estimated in our study, for both children (4–11% lower) and older adults (9–15% lower). No previous study described the most common serotypes beyond those included in PCV24.

We did not make region-stratified estimates due to sparse data, but we found regional variation in serotype distribution to be less apparent following PCV10 or PCV13 use compared with before any PCV use.^2^ Additional years of data will be needed to assess persistence of serotype-specific variation in remaining top serotypes between sites, which occurred even within regions. This could reflect annual and statistical variation and not true geographical or setting differences, but it implies these global meta-results might not reflect the experience for any particular site.^28^ Continued surveillance is also needed to affirm sustained low proportions of disease caused by vaccine serotypes. Furthermore, although thorough efforts were made to identify and include IPD data from settings with high disease burden, low-income countries, and 3 + 0 schedules to adequately assess the degree of heterogeneity globally, data were sparse.^18^ The four African meningitis belt countries with eligible data (The Gambia, Cameroon, Benin, and Kenya) did not conduct national surveillance and thus captured few cases in the mature PCV10 or PCV13 period (ranging from two to 25 cases for children aged <5 years and two to four cases for adults aged ≥50 years). Published estimates of the serotype distribution of pneumococcal meningitis cases following PCV13 introduction in Burkina Faso, Niger, Ghana, Cameroon, and Benin indicated a high proportion of vaccine-type disease (29–45% among children aged <5 years and 53–74% among those aged ≥5 years), with more than half due to serotype 1, which is a frequent cause of meningitis outbreaks. However, these analyses included data from early years following PCV13 introduction and thus did not meet eligibility criteria for inclusion in our analysis.^29–33^ A serotype 1 analysis of the PSERENADE data, which only had data from non-meningitis belt countries, showed outbreaks persisted for up to 4 years after PCV10 or PCV13 introduction but declined after 6 years in all ages by 95%.^34^ Continued monitoring is needed to understand the effect of PCVs on serotype 1 in high-burden settings.

Newer PCVs are starting to replace Synflorix and Prevenar 13. Pneumosil, a ten-valent PCV that includes serotypes 19A and 6A but not serotypes 4 and 18C, which are included in Synflorix and Prevenar 13, provides a more affordable option relative to other PCVs for countries ineligible for support from Gavi, the Vaccine Alliance. We were unable to estimate the serotype coverage of Pneumosil in mature PCV10 and PCV13 settings because we do not know whether incidence due to non-vaccine types 4 and 18C should reflect pre-PCV prevalence or increase similarly to other non-vaccine types.^21^ We estimated that PCV15 has the potential to target up to an additional 5 percentage points of disease beyond PCV13 at PCV10 sites and an additional 10 percentage points at PCV13 sites for both children and adults. PCV20 targets about half of all non-PCV13-type disease. However, the real-life impact of PCV15 and PCV20 is awaited because the benefits of higher serotype coverage could be offset by lower immunogenicity to most PCV10 and PCV13 serotypes.^35,36^ PCV24 and PCV25 products targeted at infants are in preclinical or phase 1 or 2 trials.^13–16^ PCV24 targets an additional 2–4% of IPD beyond PCV20 among children and 6–9% among adults, whereas PCV25 targets 11–16% of IPD beyond PCV24 among children and 6–9% more among adults.

PCV21 (CAPVAXIVE), targeted at adults,^12^ predominantly covers serotypes not included in PCV10 or PCV13 and includes seven serotypes not in PPV23, several of which were in the top-ten non-PCV13 types in both adults and children. We estimated that PCV21 targets approximately 62% of all adult IPD beyond PCV13 types in mature PCV13 settings and 46% in mature PCV10 settings (and in children aged <5 years, 61% in mature PCV13 settings and 38% in mature PCV10 settings), and 20–27% of adult IPD beyond that targeted by PCV20 (appendix 2 pp 14–15). Thus, a PCV21 adult immunisation programme in conjunction with infant vaccination using a different PCV has the potential to substantially reduce disease in adults if the vaccine-type effect from PCV21 is similar to that seen with PCV10 and PCV13 for children.^21^ Although we see herd protection in adults by immunising children with PCVs, it is unclear whether children can be similarly protected by immunising adults with PCVs.

Some vaccine types appear harder to eliminate than others. Serotypes 19A, 19F, 14, and 7F were reduced but persisted in both children and adults despite 7 years or more of PCVs targeting these serotypes. For countries wishing to reduce the number of doses (eg, switch to a 1 + 1 schedule), these persistent serotypes could have implications regarding how long to use the full schedule before dropping a dose, because near elimination of vaccine types is advised to prevent rebound.^37^ Serotypes differ in the amount of antibody required to prevent disease and carriage.^38^ Immunogenicity results for PCV15 and PCV20 suggest that non-inferiority criteria are only met for certain serotypes after four doses.^39,40^ It is unclear whether direct and indirect protection against serotypes in common will be equivalent between higher-valency PCVs and PCV10 or PCV13.

Limitations of this global analysis, in addition to those described above, include the inability to estimate the serotype distribution by paediatric immunisation schedule. Few sites using 3 + 0 schedules were included, and generally they had few cases. Because most sites with 3 + 1 schedules were from North America, whereas most with 2 + 1 schedules were from Europe, comparisons between them would be confounded by setting. Our results reflect primarily 2015–18 (ie, before the COVID-19 pandemic) and additional reduction of vaccine types might have occurred. Furthermore, IPD incidence was temporarily drastically reduced during the height of COVID-19 mitigation measures, and the longer-term effects of that interruption are unknown.^41–43^ Immunity gaps are likely to have also occurred during the COVID-19 pandemic period due to children missing vaccinations,^3^ which could have increased the proportion of IPD due to vaccine types. However, analyses from England, Germany, and Israel found that the serotype distribution of IPD in the COVID-19 era did not meaningfully change (comparing 2020–21 with previous years).^42–44^ If this holds true globally, the results of this analysis are likely to still be representative of the current setting.

In conclusion, new and upcoming PCVs could make further inroads beyond PCV10 and PCV13 in reducing IPD by targeting important remaining serotypes. However, for global impact they must be affordable, sufficiently immunogenic, target important remaining serotypes in all regions, and have greater vaccine-type IPD reductions than non-vaccine-type replacements. Because few data were available from high-burden settings, assessing the added value of higher-valency PCVs on already highly effective PCV10 and PCV13 programmes in these settings requires more data. Serotype 19A is the dominant serotype at PCV10 sites, causing almost one-third of disease among children, and is targeted by all other products, including Pneumosil (a ten-valent PCV) and Inventprise (a 25-valent PCV), which could be more affordable options for many low-income and middle-income countries. However, the impact of switching from Synflorix or Prevenar 13 to Pneumosil remains to be seen but might be equivalent to Synflorix if serotypes 4 and 18C return and expand like other non-vaccine types. New technology will be needed to address serotype 3, which was a top serotype in all settings.^21,38^ Because we did not identify any dominant non-PCV13-type serotypes, eliminating remaining IPD will be difficult with vaccines targeting portions of remaining serotypes. However, several higher-valency PCVs targeted at children cover more than half of remaining IPD cases and might affect disease in all ages. PCV21 is currently targeted only at adults and might reduce IPD in adults; whether indirect effects in children will occur is unknown, but most high-burden countries will probably not be able to afford an adult immunisation programme nor have existing infrastructure to execute one. It is especially important for global equity that children in the poorest settings where incidence is highest have access to higher-valency products if they prove effective in preventing remaining pneumococcal disease.

## Supplementary Material

Supplement 1

Supplement 2

## Figures and Tables

**Table:** Sites included in analysis by product and age group

	Children aged <5 years					Adults aged ≥50 years				
	Number of sites with eligible data	Number of sites with <20 cases	Total eligible IPD cases	Median PCV coverage*	Number of sites that used PCV7†	Number of sites with eligible data	Number of sites with <20 cases	Total eligible IPD cases	Median PCV coverage*	Number of sites that used PCV7†
**PCV10 sites**										
Total	12	7 (58%)	1109	93% (89–94)	2 (17%)	11	2 (18%)	3978	92% (90–95)	3 (27%)
UN region										
Europe	5 (42%)	3 (60%)	127	93% (89–94)	2 (40%)	5 (45%)	0	2490	93% (90–93)	2 (40%)
North America	0	··	··	··	··	0	··	··	··	··
Latin America and Caribbean	4 (33%)	1 (25%)	962	93% (90–94)	0	4 (36%)	0	1485	91% (89–93)	1 (25%)
Sub-Saharan Africa	2 (17%)	2 (100%)	8	87% (86–88)	0	1 (9%)	1 (100%)	2	88% (88–88)	0
Northern Africa and Western Asia	1 (8%)	1 (100%)	12	100%	0	1 (9%)	1 (100%)	1	100%	0
Asia	0	··	··	··	··	0	··	··	··	··
Oceania	0	··	··	··	··	0	··	··	··	··
Infant schedule										
2+1	8 (67%)	3 (38%)	1077	94% (93–97)	2 (25%)	8 (73%)	1 (13%)	3926	93% (92–97)	2 (25%)
3+1	1 (8%)	1 (100%)	6	88%	0	1 (9%)	0	28	90%	0
3+0	3 (25%)	3 (100%)	26	84% (84–87)	0	2 (18%)	1 (50%)	24	86% (85–87)	1 (50%)
**PCV13 sites**										
Total	42 (100%)	13 (31%)	7318	93% (87–95)	35 (83%)	31 (100%)	3 (10%)	43 774	90% (86–95)	27 (87%)
UN region										
Europe	13 (31%)	1 (8%)	4113	93% (90–94)	13 (100%)	12 (39%)	0	34 492	92% (90–94)	12 (100%)
North America	10 (24%)	1 (10%)	1134	88% (84–96)	10 (100%)	8 (26%)	1 (13%)	5935	82% (80–88)	8 (100%)
Latin America and Caribbean	8 (19%)	6 (75%)	382	93% (88–97)	4 (50%)	5 (16%)	1 (20%)	364	94% (89–97)	2 (40%)
Sub-Saharan Africa	5 (12%)	3 (60%)	632	73% (73–81)	2 (40%)	2 (6%)	1 (50%)	759	83% (77–89)	1 (50%)
Northern Africa and Western Asia	1 (2%)	0	278	94%	1 (100%)	1 (3%)	0	702	94%	1 (100%)
Asia	3 (7%)	1 (33%)	217	98% (93–98)	3 (100%)	2 (6%)	0	351	93% (91–96)	2 (100%)
Oceania	2 (5%)	1 (50%)	562	93% (93–93)	2 (100%)	1 (3%)	0	1171	94% (94–94)	1 (100%)
Infant schedule										
2+1	25 (60%)	6 (24%)	5659	93% (88–94)	23 (92%)	22 (71%)	0	37 655	91% (88–94)	20 (91%)
3+1	10 (24%)	2 (20%)	1070	93% (87–98)	10 (100%)	6 (19%)	1 (17%)	4942	85% (81–94)	6 (100%)
3+0	7 (17%)	5 (71%)	589	89% (77–94)	2 (29%)	3 (10%)	2 (67%)	1177	94% (92–94)	1 (33%)

Data are n, n (%), or median (IQR). UN regions are Europe, North America, Latin America and Caribbean, Sub-Saharan Africa, Northern Africa and Western Asia, Asia, and Oceania. Additional data are presented in appendix 2 (pp 20–23). IPD=invasive pneumococcal disease. PCV=pneumococcal conjugate vaccine. PCV7=seven-valent PCV (Prevenar, Pfizer). PCV10=ten-valent PCV (Synflorix, GSK). PCV13=13-valent PCV (Prevenar 13, Pfizer).

* The average infant PCV coverage was estimated for each site in the years eligible for the respective analysis, which differed by age group.

† Number of sites where PCV7 was used in the infant immunisation programme before PCV10 or PCV13 introduction.

## Data Availability

Restrictions apply to the availability of these data. Data were obtained under data sharing agreements from contributing surveillance sites and can only be shared by contributing organisations with their permission.

## Research in context

### Evidence before this study

Many countries have published country-specific serotype distributions of invasive pneumococcal disease (IPD) after introducing ten-valent or 13-valent pneumococcal conjugate vaccines (PCV10 or PCV13) into their national immunisation programmes. On Aug 23, 2024, we searched PubMed without language restrictions for global analyses of IPD serotype distribution following PCV10 or PCV13 introduction published between Jan 1, 2015, and Aug 23, 2024, using the search terms “PCV”, “IPD”, “serotype distribution”, AND “(global OR countries)”. Previous multicountry analyses were either restricted to specific regions, high-income countries, certain serotypes, or certain at-risk populations, or included IPD during early PCV10 or PCV13 use. Additionally, published results varied widely due to both site differences in PCV use (ie, number of years PCVs had been used for, dosing schedules, and previous seven-valent PCV use before switching to PCV10 or PCV13) and analytical differences (ie, different age groups and statistical methodology). Many studies published serotype distribution estimates for the entire post-PCV10 or post-PCV13 period, thereby including the early years of PCV use when the serotype distribution was shifting from vaccine-type to non-vaccine-type serotypes. Due to the variability of previous studies in timing and methodology, the post-PCV10 or post-PCV13 global serotype distribution, which is important for evaluating PCV10 and PCV13 impact and for estimating the potential effect on remaining disease of new or upcoming higher-valency PCVs, was not well defined.

### Added value of this study

This analysis sought to include all available data globally to estimate the IPD serotype distribution in mature PCV10 and PCV13 settings (ie, at least 5 years after introduction and with high PCV uptake). Site collaboration to share data enabled inclusion of unpublished data and facilitated evaluation of serotype data quality and use of statistical methods that maximised the use of partially serotyped data, enabling production of robust, standardised, age-stratified results. This analysis also described the serotype distribution for all ages, specifically for age groups younger than 2 years, 2–4 years, 5–17 years, 18–49 years, 50–64 years, 65–74 years, 75–84 years, and 85 years or older.

### Implications of all the available evidence

Global estimation of IPD serotype distribution after extensive PCV10 or PCV13 use resets the benchmark from which subsequent pneumococcal vaccines will be evaluated. Multiple new higher-valency PCVs target over half of remaining IPD serotypes and are anticipated to further reduce IPD, although their lower immunogenicity for most PCV10 and PCV13 serotypes warrants continued surveillance to determine their true value.
