# Correction to *Lancet Infect Dis* 2024; published online Dec 17. https://doi.org/10.1016/S1473-3099(24)00588-7

**DOI:** 10.1016/S1473-3099(25)00002-7

**Published:** 2025-02

*Garcia Quesada M, Peterson ME, Bennett JC, et al. Serotype distribution of remaining invasive pneumococcal disease after extensive use of ten-valent and 13-valent pneumococcal conjugate vaccines (the PSERENADE project): a global surveillance analysis.* Lancet Infect Dis *2024; published online Dec 17. https://doi.org/10.1016/S1473-3099(24)00588-7*—In this Article, Tine Dalby should have had the additional affiliation “Department of Bacteria, Parasites and Fungi, Statens Serum Institut, Copenhagen, Denmark”. This correction has been made to the online version as of Jan 7, 2025, and will be made to the printed version.

