# Supplementary material for: Serotype distribution of remaining invasive pneumococcal disease after extensive use of ten-valent and 13-valent pneumococcal conjugate vaccines (the PSERENADE project): a global surveillance analysis
Source: Lancet Infect Dis. Author manuscript; Available in PMC 2025 Apr 1. (PMC11947070; doi:10.1016/S1473-3099(24)00588-7)
Supplement: Supplement 1 [file NIHMS2058691-supplement-Supplement_1.pdf]

THE LANCET
Infectious Diseases

Supplementary appendix 1

This appendix formed part of the original submission and has been peer reviewed. We post it as supplied by the authors.

Supplement to: Garcia Quesada M, Peterson ME, Bennett JC, et al. Serotype distribution of remaining invasive pneumococcal disease after extensive use of ten-valent and 13-valent pneumococcal conjugate vaccines (the PSERENADE project): a global surveillance analysis. *Lancet Infect Dis* 2024; published online Dec 17. https://doi.org/10.1016/S1473-3099(24)00588-7.

Appendix 1

Contents

Appendix Table A1. PSERENADE Team Group Authors. 2

Appendix Table A2. Acknowledgment List. 4

Appendix Table A1. PSERENADE Team Group Authors.

Name	Affiliation
Mignon du Plessis, PhD	Centre for Respiratory Diseases and Meningitis, National Institute for Communicable Diseases of the National Health Laboratory Service, Sandringham, 2192 Johannesburg, South Africa
Linda de Gouveia, Btech	Centre for Respiratory Diseases and Meningitis, National Institute for Communicable Diseases of the National Health Laboratory Service, Sandringham, 2192 Johannesburg, South Africa
Jonathan Zintgraff, BSc	Servicio de Bacteriología Clínica, Departamento de Bacteriología, INEI - ANLIS "Dr. Carlos G. Malbrán", C1282 AFF, Buenos Aires, Argentina
Brita A. Winje, PhD	Faculty of Health Sciences, Oslo Metropolitan University, 0130 Oslo, Norway
Delphine Viriot, MPH	Santé Publique France, the French National Public Health Agency, 94410 Saint- Maurice, France
Maija Toropainen, PhD	Department of Health Security, Finnish Institute for Health and Welfare, 00271 Helsinki, Finland
Koh Cheng Thoon, MRCPCH	KK Women's and Children's Hospital, 229899, Singapore
Todd D. Swarthout, PhD	Julius Center for Health Sciences and Primary Care, University Medical Center Utrecht, Utrecht University, Utrecht, the Netherlands; Malawi Liverpool Wellcome Programme, P.O. Box 30096, Chichiri, Blantyre 3, Malawi; NIHR Mucosal Pathogens Research Unit, Research Department of Infection, Division of Infection and Immunity, University College London, London, UK
Catherine G. Sutcliffe, PhD	Johns Hopkins Bloomberg School of Public Health, Baltimore, MD 21205, United States
Shigeru Suga, PhD	Infectious Disease Center and Department of Clinical Research, National Hospital Organization Mie Hospital, Tsu, Mie 514-0125, Japan
Anneke Steens, PhD	Centre for Infectious Disease Control, National Institute for Public Health and the Environment, 3721 MA Bilthoven, The Netherlands
Juan Carlos Sanz, PhD	Laboratorio Regional de Salud Pública, Dirección General de Salud Pública, Comunidad de Madrid, 28055 Madrid, Spain
Leah J. Ricketson, MSc	Department of Pediatrics, University of Calgary, Calgary Alberta T3B 6A8, Canada
Gloria Rey-Benito, MSc	Pan American Health Organization, World Health Organization, Washington, DC 20037, United States
Eric Rafai, MBBS	Ministry of Health and Medical Services, Suva, Fiji
Rodrigo Puentes, MSc	Instituto de Salud Pública de Chile, 7780050 Santiago, Santiago Metropolitan, Chile
Marie-Cecile Ploy, PhD	University Hospital Centre Limoges, Regional Observatories for Pneumococci, 87000 Limoges, France
Tamara Pilishvili, PhD	Division of Bacterial Diseases, National Center for Immunizations and Respiratory Diseases, Centers for Disease Control and Prevention, Atlanta, GA 30329, USA
Kate Pennington, MPH	Communicable Disease Epidemiology and Surveillance Section, Office of Health Protection, Australian Government Department of Health, 2606 Canberra ACT, Australia
Stephen I. Pelton, MD	Boston University Schools of Medicine and Public Health, Boston, MA 02118, United States
Néhémie Nzoyikorera, PhD	National Reference Laboratory, Institut National de Santé Publique (INSP) du Burundi, Bujumbura, Burundi; Higher Institute of Bioscience and Biotechnology, Mohammed VI University of Sciences and Health (UM6SS), Casablanca, Morocco; Laboratory of Infectiology and Microbial Biotechnology Research, Mohammed VI Center for Research & Innovation (CM6), Rabat, Morocco
Tomoka Nakamura, MSPH	World Health Organization, 1202 Geneva, Switzerland
Jolita Mereckiene, MSc	Health Protection Surveillance Centre (HPSC), 25-27 Gardiner Street Middle, Dublin 1, D01 A4A3, Ireland
Ioanna Magaziotou, PhD	National Public Health Organisation, 15123 Athens, Greece
Laura MacDonald, MSc	Public Health Scotland, Glasgow, United Kingdom
Tiia Lepp, MD	Department of Communicable Disease and Control and Health Protection, Public Health Agency of Sweden, 171 82 Solna, Sweden
Geneviève Deceuninck, PhD	Laboratoire de Santé Publique du Québec, Sainte-Anne-de-Bellevue, Quebec H9X 3R5, Canada
Pavla Krizova, MD	National Institute of Public Health (NIPH), 100 00, Praha 10, Czech Republic
Vicki Krause, MD	Centre for Disease Control, Department of Health and Community Services, Darwin City NT 8000, Australia

Miwako Kobayashi, MD	Division of Bacterial Diseases, National Center for Immunizations and Respiratory Diseases, Centers for Disease Control and Prevention, Atlanta, GA 30329, USA
Aníbal Kawabata, BSc	Laboratorio Central de Salud Pública, Asunción, Paraguay (Central Laboratory of Public Health, Asunción, Paraguay), Asunción, Paraguay
Ilias Hossain, MSc	Medical Research Council Unit The Gambia at London School of Hygiene & Tropical Medicine, PO Box 273, Banjul, The Gambia
Marcela Guevara, MD	Instituto de Salud Pública de Navarra - IdiSNA, 31003 Pamplona, Navarra, Spain; CIBER Epidemiología y Salud Pública, (CIBERESP), 28029 Madrid, Spain
Theano Georgakopoulou, MD	Department for Vaccine Preventable Diseases, National Public Health Organization, 15123 Athens, Greece
Yvonne Galloway, BSc	Epidemiology Team, Institute of Environmental Science and Research, Porirua, 5022 Wellington, New Zealand
Helga Erlendsdottir, MS	Department of Clinical Microbiology, Landspitali - The National University Hospital, Hringbraut, 101 Reykjavik, Iceland
Janepsy Díaz, PhD	Instituto de Salud Pública de Chile, 7780050 Santiago, Santiago Metropolitan, Chile
Elina Dimina, MD	Centre for disease prevention and control of Latvia, Riga, 1005, Latvia
Martina Del Manso, MD	Department of Infectious Diseases, Italian National Institute of Health (Istituto Superiore di Sanità, ISS), 00161 Rome, Italy
Geneviève Deceuninck, MD	Quebec University Hospital Research Centre, Québec, QC G1V 4G2, Canada
Pilar Ciruela, MD, PhD	Surveillance and Public Health Emergency Response, Public Health Agency of Catalonia, 08005 Barcelona, Spain; CIBER Epidemiología y Salud Pública, (CIBERESP), 28029 Madrid, Spain
Kin-Hung Chow, MSc	Department of Microbiology and Carol Yu Centre for Infection, Queen Mary Hospital, The University of Hong Kong, Hong Kong SAR, China
Guanhao Chan, MSc	Singapore Ministry of Health, Communicable Diseases Division, 169854, Singapore
Gustavo Chamorro, BSc	National Program on Immunopreventable Diseases and Expanded Program on Immunizations, Asunción, Paraguay
Lucia Pastore Celentano, MD	European Centre for Disease Prevention and Control, 169 73 Solna, Sweden
Carrie L. Byington, MD	University of Utah Department of Pediatrics (emeritus), Salt Lake City, UT 84108, United States; University of California Health System, Oakland, CA 94607, United States
Dana Bruden, MS	Arctic Investigations Program, Division of Preparedness and Emerging Infections, National Center for Emerging and Zoonotic Infectious Diseases, Centers for Disease Control and Prevention, Anchorage, AK 99508, United States
Maria-Cristina C. Brandileone, PhD	National Laboratory for Meningitis and Pneumococcal Infections, Center of Bacteriology, Institute Adolfo Lutz (IAL), São Paulo, 01246-902, Brazil
Godfrey Bigogo, PhD	Centre for Global Health Research, Kenya Medical Research Institute, P.O. Box: 1578 - 40100, Kisumu, Kenya
Mária Avdičová, MD	National Reference Centre for Pneumococcal and Haemophilus Diseases, Regional Authority of Public Health, 975 56 Banská Bystrica, Slovak Republic
Laurie Aukes, RN	Vaccine Study Center, Kaiser Permanente, Oakland, CA 94612, United States
Zahin Amin-Chowdhury, MSc	Immunisation and Countermeasures Division, Public Health England, NW9 5EQ, London, United Kingdom
WHO Invasive Bacterial Vaccine-Preventable Diseases (IB-VPD) Network	World Health Organization, 1202 Geneva, Switzerland
Toronto Invasive Bacterial Diseases Network	Toronto Invasive Bacterial Diseases Network, and Department of Laboratory Medicine and Pathobiology, University of Toronto, Toronto, ON M5S 1A8 Canada

Appendix Table A2. Acknowledgment List.

**PSERENADE Technical Advisory Group**
Thomas Cherian
William P. Hausdorff
Marc Lipsitch
Shabir A. Madhi
Elizabeth Miller
Catherine Satzke
Cynthia G. Whitney
Ron Dagan
**World Health Organization**
Jenny A. Walldorf
Heidi Soeters
**Johns Hopkins University**
Yunfeng Cao
Peggy Gross
Carly Herbert
Donna Hesson
Ananya Kumar
Anju Ogyu
Kate Perepezko
E. Wangeci Kagucia
Francesca Schiaffino Salazar
Jenna Sinkevitch
Daniel Stephens
Melody Xiao
**EpiConcept**
Germaine Hanquet
**Epidemiology Department, Dirección General de Salud Pública, Comunidad de Madrid, Madrid, Spain**
Luis García Comas
Maria Ordobás Gavín
**Department of Infectious Diseases, Italian National Institute of Health (Istituto Superiore di Sanità, ISS), Rome, Italy**
Flavia Riccardo
**Surveillance and Public Health Emergency Response, Public Health Agency of Catalonia, Barcelona, Spain**
Sonia Broner
Conchita Izquierdo
**Institut de Recerca Sant Joan de Déu, Hospital Sant Joan de Deu, Barcelona, Spain**
Desiree Henares
**Department of Microbiology, Faculty of Medicine and Pharmacy, Hassan II University of Casablanca, Casablanca, Morocco**
Khalid Zerouali
**Laboratoire du Centre Mère et Enfant Fondation Chantal Biya, Yaoundé, Cameroun**
Angeline Boula
**University of Parakou, Alibori in PARAKOU, Benin**
Joseph Agossou
**WHO Collaborating Centre for New Vaccines Surveillance, Medical Research Council Unit The Gambia at London School of Hygiene & Tropical Medicine, Banjul, The Gambia**
Brenda A. Kwambana-Adams
Martin Antonio
Archibald Worwui
Peter S. Ndow
**WHO Regional Office for Africa, Inter Country Support Team, Ouagadougou, Burkina Faso**
Joseph Biey
Bernard Ntsama
**WHO Regional Office for Africa, Inter Country Support Team, Libreville, Gabon**
Gilson Paluku
Aboubacar N'DIAYE
**WHO Country offices, Benin and Cameroon**
**Sentinel site surveillance teams and countries part of the African Pediatric Bacterial Meningitis Surveillance Network**
**Australian National Notifiable Diseases Surveillance data were provided by the Office of Health Protection, Australian Government Department of Health, on behalf of the Communicable Diseases Network Australia and the Enhanced Invasive Pneumococcal Disease Surveillance Working Group.**
