# Supplementary material for: Serotype distribution of remaining invasive pneumococcal disease after extensive use of ten-valent and 13-valent pneumococcal conjugate vaccines (the PSERENADE project): a global surveillance analysis
Source: Lancet Infect Dis. Author manuscript; Available in PMC 2025 Apr 1. (PMC11947070; doi:10.1016/S1473-3099(24)00588-7)

THE LANCET
Infectious Diseases

Supplementary appendix 2

This appendix formed part of the original submission and has been peer reviewed. We post it as supplied by the authors.

Supplement to: Garcia Quesada M, Peterson ME, Bennett JC, et al. Serotype distribution of remaining invasive pneumococcal disease after extensive use of ten-valent and 13-valent pneumococcal conjugate vaccines (the PSERENADE project): a global surveillance analysis. *Lancet Infect Dis* 2024; published online Dec 17. https://doi.org/10.1016/S1473-3099(24)00588-7.

Appendix 2 for Global Serotype Distribution of Remaining Invasive Pneumococcal Disease After Extensive Use of PCV10/13: The PSERENADE Project

Contents

Supplementary Methods 2

Supplementary Tables 10

Table S1. Serotypes included in current and upcoming PCV products. 10

Table S2. Individual site characteristics during the years that met the criteria for mature PCV10 or PCV13 years included in the analysis. 11

Table S3. Percentage of IPD cases in the mature PCV10/13 period due to serotypes included in current and upcoming products, corresponding to Figure 2. 14

Table S4. Serotype-specific distribution of IPD in the mature PCV10/13 period, corresponding to Figure 3. 15

Table S5. Percentage of IPD cases in the mature PCV13 period due to serotypes included in current and upcoming products, stratified by finer age groups. 16

Table S6. Serotype-specific distribution of IPD in the mature PCV13 period, stratified by finer age groups (corresponds to Figure S5). 17

Table S7. Percent of IPD due to serotypes included in current and upcoming PCVs, by region, corresponding to data shown in Figure 4. 18

Table S8 (A-B). Summary of sites included in analysis by product and age group, showing additional stratifications to those in Table 1. 20

Supplementary Figures 24

Figure S1 (A-B). Serotype distribution within the subgroup of PCV13-related serotypes and subgroup of non-PCV13-related serotypes. 24

Figure S2. Serotype-specific distribution of IPD in the mature PCV10/13 period, as shown in Figure 3, but with serotypes colored by inclusion in PCV21 or PCV25. 26

Figure S3 (A-B). Age distribution of IPD cases at PCV13 sites included in age-stratified analyses, ordered by number of serotyped cases included. 27

Figure S4 (A-B). Serotype-specific distribution of IPD in the mature PCV13 period, stratified by finer age groups. 29

Figure S5 (A-D). Site-specific rank and proportion of IPD due to top 6 serotypes from global meta-average (Figure 2/Table S4) for each age and product strata, colored by region. 30

Supplementary Methods

The following R code was used to implement the multinomial Dirichlet regression model described in the manuscript. The output is an estimate of the distribution of vaccine-type categories (e.g., % PCV10-type, % PCV13-type, etc.) and of individual serotypes (e.g., % serotype 3) across sites included in the analysis, with 95% confidence intervals. Data was structured as one row per site and year of data, with variables for IPD case counts for vaccine-type and non-vaccine-type categories (e.g., `pcv10`, `non_pcv10`, etc.) as well as individual serotypes (e.g., `st3`, `st4`, etc.). Analyses generated separate estimates for PCV10 vs PCV13-using sites, as defined by the `product` variable. Similarly, code was adapted to estimate serotype distribution for finer age groups in PCV13 sites by using a variable for age group instead.

```
################################################################################
#################### Frequently used functions #################################
################################################################################

# Libraries ----
library(dplyr)
library(VGAM)
library(mgcv)
library(matrixStats)
library(boot)
library(resample)

# Function for summarizing case counts for the model ----
f.sum.data <- function(df,...) {
  df2 <- df %>%
    select(site,product,region,...) %>%
    group_by(site,product,region) %>%
    summarise_all(funs(sum)) %>%
    ungroup() %>%
    mutate(total = rowSums(.[4:ncol(.)])) %>%
    arrange(product)

  # drop sites with no other top st cases
  df2<-subset(df2,df2$total>0)
}

# Function for running the model----
f.prd <- function(...,.df,st.names,boot="no",coef="no",coef.x=NA){

  prd.df <- .df %>%
    group_by(product) %>%
    filter(n()>1) %>%
    arrange(product)

  prd.label <- unique(prd.df$product)

  if (length(unique(prd.df$product)) == 1) {
    if (boot == "no") {
      attach(prd.df)
      vglm.prd <- vglm(cbind(...) ~ 1,
                       dirmultinomial(lphi = "logit", iphi = 0.1, parallel = FALSE,
zero = "M"),
                       data = prd.df, trace = TRUE)
      res.prd <- uniquecombs(round(predictvglm(vglm.prd, type="response"),10))
      detach(prd.df)
    }

    if (boot == "yes") {
      attach(prd.df)
      vglm.prd <- vglm(cbind(...) ~ 1,
```

```
                        dirmultinomial(lphi = "logit", iphi = 0.1, parallel = FALSE,
zero = "M"),
                        data = prd.df, trace = TRUE, coefstart = coef.x, maxit = 1)
      res.prd <- uniquecombs(round(predictvglm(vglm.prd, type="response"),10))
      detach(prd.df)
    }

    if (coef == "no"){
      res.prd <- matrix(res.prd,nrow = 1, ncol = length(res.prd))
      colnames(res.prd)<-st.names
      rownames(res.prd)<-prd.label

      return(res.prd)
    }

    if (coef == "yes") {
      coef.all<- coefficients(vglm.prd)
      return(coef.all)
    }

  } else {
    if (boot == "no") {
      attach(prd.df)
      vglm.prd <- vglm(cbind(...) ~ 1 + product,
                        dirmultinomial(lphi = "logit", iphi = 0.1, parallel = FALSE,
zero = "M"),
                        data = prd.df, trace = TRUE)
      res.prd <- uniquecombs(round(predictvglm(vglm.prd, type="response"),10))
      detach(prd.df)
    }

    if (boot == "yes") {
      attach(prd.df)
      vglm.prd <- vglm(cbind(...) ~ 1 + product,
                        dirmultinomial(lphi = "logit", iphi = 0.1, parallel = FALSE,
zero = "M"),
                        data = prd.df, trace = TRUE, coefstart = coef.x, maxit = 1)
      res.prd <- uniquecombs(round(predictvglm(vglm.prd, type="response"),10))
      detach(prd.df)
    }

    if (coef == "no"){
      res.prd <- matrix(res.prd,nrow = nrow(res.prd), ncol = ncol(res.prd))
      colnames(res.prd)<-st.names
      rownames(res.prd)<-prd.label

      return(res.prd)
    }

    if (coef == "yes") {
      coef.all<- coefficients(vglm.prd)
      return(coef.all)
    }
  }
}

################################################################################
############# Estimating % VT for different products ##########################
################################################################################

# Run regressions outside the bootstrap to get initial coefficients ----
# each of these are used to estimate %PCV10, %PCV13, etc.
coef.pcv10 <- f.prd(pcv10,non_pcv10,.df = pser.pcv10.df,
```

```
                    st.names = c("PCV10","non-PCV10"),
                    coef = "yes")

coef.pcv13 <- f.prd(pcv13,non_pcv13,.df = pser.pcv13.df,
                    st.names = c("PCV13","non-PCV13"),
                    coef = "yes")

coef.pcv15 <- f.prd(pcv15,non_pcv15,.df = pser.pcv15.df,
                    st.names = c("PCV15","non-PCV15"),
                    coef = "yes")

coef.pcv20 <- f.prd(pcv20,non_pcv20,.df = pser.pcv20.df,
                    st.names = c("PCV20","non-PCV20"),
                    coef = "yes")

coef.pcv24 <- f.prd(pcv24,non_pcv24,.df = pser.pcv24.df,
                    st.names = c("PCV24","non-PCV24"),
                    coef = "yes")

coef.ppv23 <- f.prd(ppv23,non_ppv23,.df = pser.ppv23.df,
                    st.names = c("PPV23","non-PPV23"),
                    coef = "yes")

coef.pcv21 <- f.prd(pcv21,non_pcv21,.df = pser.pcv21.df,
                    st.names = c("PCV21","non-PCV21"),
                    coef = "yes")

coef.pcv21or13 <- f.prd(pcv21or13,non_pcv21or13,.df = pser.pcv21or13.df,
                        st.names = c("PCV21or13","non-PCV21or13"),
                        coef = "yes")

coef.pcv21non13 <- f.prd(pcv21non13,non_pcv21non13,.df = pser.pcv21non13.df,
                         st.names = c("PCV21non13","non-PCV21non13"),
                         coef = "yes")

coef.pcv25 <- f.prd(pcv25,non_pcv25,.df = pser.pcv25.df,
                    st.names = c("PCV25","non-PCV25"),
                    coef = "yes")

# Function to bootstrap model with coefficient for product (PCV10 sites vs PCV13
sites) ----
f.vglm.stcat <- function(d, i){
  d2 <- d[i,]

  # summarize data for pcv10, 13, 15, 20, 24, 23 vs non ----
  pser.pcv10.df <- f.sum.data(d2,pcv10,non_pcv10)
  pser.pcv13.df <- f.sum.data(d2,pcv13,non_pcv13)
  pser.pcv15.df <- f.sum.data(d2,pcv15,non_pcv15) %>%
    filter(site %notin% sites_pcv13_only) # filter out sites that can only identify
PCV13-types
  pser.pcv20.df <- f.sum.data(d2,pcv20,non_pcv20) %>%
    filter(site %notin% sites_pcv13_only) # filter out sites that can only identify
PCV13-types
  pser.pcv24.df <- f.sum.data(d2,pcv24,non_pcv24) %>%
    filter(site %notin% sites_pcv13_only) # filter out sites that can only identify
PCV13-types
  pser.ppv23.df <- f.sum.data(d2,ppv23,non_ppv23) %>%
    filter(site %notin% sites_pcv13_only) # filter out sites that can only identify
PCV13-types
  pser.ppv23.df <- f.sum.data(d2,ppv23,non_ppv23) %>%
    filter(site %notin% sites_pcv24_only) # filter out sites that can only identify
PCV24-types
  pser.pcv21.df <- f.sum.data(d2,pcv21,non_pcv21) %>%
```

```
    filter(site %notin% sites_pcv24_only) # filter out sites that can only identify
PCV24-types
  pser.pcv25.df <- f.sum.data(d2,pcv25,non_pcv25) %>%
    filter(site %notin% sites_pcv24_only) # filter out sites that can only identify
PCV24-types

  # regressions ----

  pcv10.prd <- f.prd(pcv10,non_pcv10,.df = pser.pcv10.df,
                     st.names = c("PCV10","non-PCV10"),
                     boot = "yes",coef.x = coef.pcv10)

  pcv13.prd <- f.prd(pcv13,non_pcv13,.df = pser.pcv13.df,
                     st.names = c("PCV13","non-PCV13"),
                     boot = "yes",coef.x = coef.pcv13)

  pcv15.prd <- f.prd(pcv15,non_pcv15,.df = pser.pcv15.df,
                     st.names = c("PCV15","non-PCV15"),
                     boot = "yes",coef.x = coef.pcv15)

  pcv20.prd <- f.prd(pcv20,non_pcv20,.df = pser.pcv20.df,
                     st.names = c("PCV20","non-PCV20"),
                     boot = "yes",coef.x = coef.pcv20)

  pcv24.prd <- f.prd(pcv24,non_pcv24,.df = pser.pcv24.df,
                     st.names = c("PCV24","non-PCV24"),
                     boot = "yes",coef.x = coef.pcv24)

  ppv23.prd <- f.prd(ppv23,non_ppv23,.df = pser.ppv23.df,
                     st.names = c("PPV23","non-PPV23"),
                     boot = "yes",coef.x = coef.ppv23)

  pcv21.prd <- f.prd(pcv21,non_pcv21,.df = pser.pcv21.df,
                     st.names = c("PCV21","non-PCV21"),
                     boot = "yes",coef.x = coef.pcv21)

  pcv25.prd <- f.prd(pcv25,non_pcv25,.df = pser.pcv25.df,
                     st.names = c("PCV25","non-PCV25"),
                     boot = "yes",coef.x = coef.pcv25)

  # calculate %s for each ----

  figure.matrix <- cbind(pcv10.prd[,1],
                         pcv13.prd[,1],
                         pcv15.prd[,1],
                         pcv20.prd[,1],
                         pcv24.prd[,1],
                         ppv23.prd[,1],
                         pcv21.prd[,1],
                         pcv25.prd[,1])

  colnames(figure.matrix) <- c("PCV10","PCV13", "PCV15", "PCV20",
                               "PCV24", "PPV23","PCV21", "PCV25")
  figure.matrix <- 100*figure.matrix

  #PCV10 then PCV13 sites
  vector.prd <- c(figure.matrix[1,],figure.matrix[2,])
  # return ----
  return(vector.prd)

}

# Run the bootstrap with stratified resampling by PCV10 vs PCV13 sites ----
```

```
set.seed(159)
pserenade.boot.stcat <- boot(data = pser.site, statistic = f.vglm.stcat,
                             strata = pser.site$product, R = 100,
                             parallel = "multicore")

boot.stcat.means <- colMeans(pserenade.boot.stcat$t)
boot.stcat.medians <- colMedians(pserenade.boot.stcat$t)
boot.stcat.observed <- pserenade.boot.stcat$t0

boot.stcat.matrix <-
matrix(cbind(boot.stcat.observed,boot.stcat.means,boot.stcat.medians,NA,NA),
                            nrow = 16,ncol = 5,dimnames = list(c("PCV10 - PCV10",
                                                                 "PCV10 - PCV13",
                                                                 "PCV10 - PCV15",
                                                                 "PCV10 - PCV20",
                                                                 "PCV10 - PCV24",
                                                                 "PCV10 - PPV23",
                                                                 "PCV10 - PCV21",
                                                                 "PCV10 - PCV25",
                                                                 "PCV13 - PCV10",
                                                                 "PCV13 - PCV13",
                                                                 "PCV13 - PCV15",
                                                                 "PCV13 - PCV20",
                                                                 "PCV13 - PCV24",
                                                                 "PCV13 - PPV23",
                                                                 "PCV13 - PCV21",
                                                                 "PCV13 -
PCV25"),c("Observed", "Boot mean", "Boot median",

"Lower 95%CI", "Upper 95%CI")))


for(i in 1:16){
  bca.ci <- boot.ci(boot.out = pserenade.boot.stcat, type = c("bca"), index=i)
  boot.stcat.matrix[i,4] <- bca.ci$bca[,4]
  boot.stcat.matrix[i,5] <- bca.ci$bca[,5]
}

boot.stcat.data <- data.frame(observed = boot.stcat.matrix[,1],
                              mean = boot.stcat.matrix[,2],
                              ci.low = boot.stcat.matrix[,4],
                              ci.high = boot.stcat.matrix[,5])

boot.stcat.data$vaccine <- substring(rownames(boot.stcat.data),first = 9)
boot.stcat.data$vaccine <- factor(boot.stcat.data$vaccine, levels =
c("PCV10","PCV13","PCV15","PCV20","PCV24","PPV23"))
boot.stcat.data$site.product <- substring(rownames(boot.stcat.data),first = 1, last =
5)
boot.stcat.data$site.product <- factor(boot.stcat.data$site.product,levels =
c("PCV10","PCV13"))

################################################################################
############# Estimating % due to individual serotypes ########################
################################################################################

# Run additional regressions outside the bootstrap to get initial coefficients ----
# These are used to estimate % due to individual serotypes within a subset of IPD.
# For example, the first is used to estimate proportion of PCV7-type IPD due to
# each of the individual serotypes in PCV7.
coef.pcv7 <- f.prd(st4,st6B,st9V,st14,st18C,st19F,st23F,.df = pser.pcv7.df,
                   st.names = c("4","6B","9V","14","18C","19F","23F"),
                   coef = "yes")
```

```
coef.pcv10x <- f.prd(st1,st5,st7F,.df = pser.pcv10x.df,
                     st.names = c("1","5","7F"),
                     coef = "yes")

coef.pcv13x <- f.prd(st3,st6A,st19A,.df = pser.pcv13x.df,
                     st.names = c("3","6A","19A"),
                     coef = "yes")

coef.nvt <- f.prd(pcv15x,pcv20x,pcv24x,non_pcv24,.df = pser.nvt.df,
                  st.names = c("PCV15x", "PCV20x","pcv24x","Other"),
                  coef = "yes")

coef.pcv15x <- f.prd(st22F,st33F,.df = pser.pcv15x.df,
                     st.names = c("22F","33F"),
                     coef = "yes")

coef.pcv20x <- f.prd(st8,st10A,st11A,st12F,st15BC,.df = pser.pcv20x.df,
                     st.names = c("8","10A","11A","12F","15BC"),
                     coef = "yes")

coef.pcv24x <- f.prd(st2,st9N,st17F,st20A_20B_und,.df = pser.pcv24x.df,
                     st.names = c("2","9N","17F","20"),
                     coef = "yes")

coef.other.topst <- f.prd(topst1,topst2,other,.df = pser.other.df,
                          st.names = c("Top St1","Top St2","Other"),
                          coef = "yes")

coef.top1.st <- f.prd(st13,st21,st7C,st38,st15A,st16F,st18A,st23A,st34,.df =
pser.topprd1.df,
                      st.names = c("13","21","7C","38","15A","16F","18A","23A","34"),
                      coef = "yes")

coef.top2.st <- f.prd(st23B,st24F,st25A,st28A,st31,st35B,st35F,st6C,.df =
pser.topprd2.df,
                      st.names = c("23B","24F","25A","28A","31","35B","35F","6C"),
                      coef = "yes")

# Function to jackknife model with coefficient for product (PCV10 sites vs PCV13
sites) ----
# NOTE: Jackknife used instead of bootstrap due to many 0 case counts for individual
# serotypes within certain sites, causing bootstrap to fail.
f.jack.vglm <- function(d2){

  # summarize data for pcv7, pcv10x, pcv13x, and nvt categories ----
  pser.all.df <- f.sum.data(d2,pcv7,pcv10x,pcv13x,non_pcv13)

  # summarize data for pcv7 serotypes ----
  pser.pcv7.df <- f.sum.data(d2,st4,st6B,st9V,st14,st18C,st19F,st23F)

  # summarize data for pcv10x serotypes ----
  pser.pcv10x.df <- f.sum.data(d2,st1,st5,st7F)

  # summarize data for pcv13x serotypes ----
  pser.pcv13x.df <- f.sum.data(d2,st3,st6A,st19A)

  # summarize data for nvt groups ----
  pser.nvt.df <- f.sum.data(d2,pcv15x,pcv20x,pcv24x,non_pcv24) %>%
    filter(site %notin% sites_pcv13_only) # filter out sites that can only identify
PCV13-types

  # summarize data for nvt serotypes: pcv15x (22F, 33F) ----
  pser.pcv15x.df <- f.sum.data(d2,st22F,st33F) %>%
```

```
    filter(site %notin% sites_pcv13_only) # filter out sites that can only identify
PCV13-types

  # summarize data for nvt serotypes: pcv20x (8, 10A, 11A, 12F, 15BC) ----
  pser.pcv20x.df <- f.sum.data(d2,st8,st10A,st11A,st12F,st15BC) %>%
    filter(site %notin% sites_pcv13_only) # filter out sites that can only identify
PCV13-types

  # summarize data for nvt serotypes: pcv24x (2, 9N, 17F, 20) ----
  pser.pcv24x.df <- f.sum.data(d2,st2,st9N,st17F,st20A_20B_und) %>%
    filter(site %notin% sites_pcv13_only) # filter out sites that can only identify
PCV13-types

  # summarize data for other top serotypes ----
  pser.topprd1.df<- f.sum.data(d2,st13,st21,st7C,st38,st15A,st16F,st18A,st23A,st34)
%>%
    filter(site %notin% sites_pcv24_only) # filter out sites that can only identify
PCV24-types
  pser.topprd2.df<- f.sum.data(d2,st23B,st24F,st25A,st28A,st31,st35B,st35F,st6C) %>%
    filter(site %notin% sites_pcv24_only) # filter out sites that can only identify
PCV24-types

  # summarize data for other top serotypes vs the rest of nvts ----
  pser.other.df <- f.sum.other(d2) %>%
    filter(site %notin% sites_pcv24_only)  # filter out sites that can only identify
PCV24-types

  # regressions ----
  all.prd <- f.prd(pcv7,pcv10x,pcv13x,non_pcv13,.df = pser.all.df,
                  st.names = c("PCV7 serotypes","PCV10x serotypes","PCV13x
serotypes","Non-PCV13 serotypes"),
                  boot = "yes",coef.x = coef.all)
  pcv7.prd <- f.prd(st4,st6B,st9V,st14,st18C,st19F,st23F,.df = pser.pcv7.df,
                   st.names = c("4","6B","9V","14","18C","19F","23F"),
                   boot = "yes",coef.x = coef.pcv7)
  pcv10x.prd <- f.prd(st1,st5,st7F,.df = pser.pcv10x.df,
                     st.names = c("1","5","7F"),
                     boot = "yes",coef.x = coef.pcv10x)
  pcv13x.prd <- f.prd(st3,st6A,st19A,.df = pser.pcv13x.df,
                     st.names = c("3","6A","19A"),
                     boot = "yes",coef.x = coef.pcv13x)
  nvt.prd <- f.prd(pcv15x,pcv20x,pcv24x,non_pcv24,.df = pser.nvt.df,
                  st.names = c("PCV15x", "PCV20x","pcv24x","Other"),
                  boot = "yes",coef.x = coef.nvt)
  pcv15x.prd <- f.prd(st22F,st33F,.df = pser.pcv15x.df,
                     st.names = c("22F","33F"),
                     boot = "yes",coef.x = coef.pcv15x)
  pcv20x.prd <- f.prd(st8,st10A,st11A,st12F,st15BC,.df = pser.pcv20x.df,
                     st.names = c("8","10A","11A","12F","15BC"),
                     boot = "yes",coef.x = coef.pcv20x)
  pcv24x.prd <- f.prd(st2,st9N,st17F,st20A_20B_und,.df = pser.pcv24x.df,
                     st.names = c("2","9N","17F","20"),
                     boot = "yes",coef.x = coef.pcv24x)
  other.topst.prd <- f.prd(topst1,topst2,other,.df = pser.other.df,
                          st.names = c("Top st1","Top st2","Other"),
                          boot = "yes",coef.x = coef.other.topst)
  top.st1.prd <- f.prd(st13,st21,st7C,st38,st15A,st16F,st18A,st23A,st34,.df =
pser.topprd1.df,
                      st.names = c("13","21","7C","38","15A","16F","18A","23A","34"),
                      boot = "yes",coef.x = coef.top1.st)
  top.st2.prd <- f.prd(st23B,st24F,st25A,st28A,st31,st35B,st35F,st6C,.df =
pser.topprd2.df,
                      st.names = c("23B","24F","25A","28A","31","35B","35F","6C"),
```

```
                       boot = "yes",coef.x = coef.top2.st)

  # calculate %s for each st ----
  ranked.prd <- cbind(top.st1.prd*other.topst.prd[,1],
                      top.st2.prd*other.topst.prd[,2],
                      other.topst.prd[,3])
  all.nvt.prd <- cbind(pcv15x.prd*nvt.prd[,1],
                       pcv20x.prd*nvt.prd[,2],
                       pcv24x.prd*nvt.prd[,3],
                       ranked.prd*nvt.prd[,4])
  matrix.prd <- cbind(pcv7.prd*all.prd[,1],
                      pcv10x.prd*all.prd[,2],
                      pcv13x.prd*all.prd[,3],
                      all.nvt.prd*all.prd[,4])
  matrix.prd <- 100*matrix.prd
  matrix.prd <- matrix.prd[,1:(ncol(matrix.prd)-1)] # remove row for "other" serotypes

  # vector with PCV10 then PCV13 sites ----

  vector.prd <- c(matrix.prd[1,],matrix.prd[2,])

  half <- length(vector.prd)/2
  vector.names <- c(paste("PCV10 -",names(vector.prd)[c(1:half)]),
                    paste("PCV13 -
",names(vector.prd)[c((half+1):length(vector.prd))]))

  names(vector.prd)<-vector.names

  # return ----
  return(vector.prd)

}
# Run the jackknife ----
set.seed(159)
jack.results <- jackknife(pser.site,f.jack.vglm)
jack.results

jack.data <- round(jack.results$stats,2)

jack.data$ci.low <- jack.data$Observed - 1.96*jack.data$SE
jack.data$ci.low <- ifelse(jack.data$ci.low < 0, 0, jack.data$ci.low)
jack.data$ci.high <- jack.data$Observed + 1.96*jack.data$SE

jack.data$serotype <- substring(rownames(jack.data),first = 9)
jack.data$site.product <- substring(rownames(jack.data),first = 1, last = 5)
jack.data$site.product <- factor(jack.data$site.product,levels = c("PCV10","PCV13"))
```

Supplementary Tables

Table S1. Serotypes included in current and upcoming PCV products.

	4	6B	9V	14	18C	19F	23F	1	5	7F	3	6A	19A	22F	33F	8	10A	11A	12F	15BC	2	9N	17F	20	6C	15A	16F	23A	23B	24F	31	35B
**PCV7 (Prevnar)**	X	X	X	X	X	X	X																									
**PCV10 (Synflorix)**	X	X	X	X	X	X	X	X	X	X																						
**PCV10 (Pneumosil)**		X	X	X		X	X	X	X	X		X	X																			
**PCV13 (Prevnar13)**	X	X	X	X	X	X	X	X	X	X	X	X	X																			
**PCV15 (VAXNEUVANCE)**	X	X	X	X	X	X	X	X	X	X	X	X	X	X	X																	
**PCV20 (Prevnar20)**	X	X	X	X	X	X	X	X	X	X	X	X	X	X	X	X	X	X	X	X												
**PCV24 (GSK, Vaxcyte, Merck)**	X	X	X	X	X	X	X	X	X	X	X	X	X	X	X	X	X	X	X	X	X	X	X	X								
**PCV25 (Inventprise)**	X	X	X	X	X	X	X	X	X	X	X		X	X	X	X	X		X	X	X	X			X	X	X			X		X
**PPV23 (Pneumovax23)**	X	X	X	X	X	X	X	X	X	X	X		X	X	X	X	X	X	X	X	X	X	X	X								
**PCV21 (V116)**										X	X	X	X	X	X	X	X	X	X	X		X	X	X		X	X	X	X	X	X	X

Table S2. Individual site characteristics during the years that met the criteria for mature PCV10 or PCV13 years included in the analysis.

				Infant immunization program			Adult immunization program[6]		Number of cases[7]			
Product[1]	Region[2]	Income level[3]	Site	Schedule[1]	PCV7 [4]	Median coverage[5]	Adult PCV13	Adult PPV23	Aged <5 years	Aged ≥50 years	Serotyping methods[8]	Years included[9]
PCV10	Europe	High income	Finland	2+1	None	96.10%	All Adults	All Adults	59	1246	PCR, Q	2016-2017
	Europe	High income	Iceland	2+1	None	89%[b]	High Risk	All Adults	3	42	L, PCR	2017-2018
	Europe	High income	Latvia	2+1	1-2 years	93.30%	None	None	1	50	Q, L, CST	2018
	Europe	High income	Netherlands	2+1	3+ years	93.90%	High Risk	High Risk	58	1124	Q, L	2015-2018
	Europe	Upper middle income	Bulgaria	3+1	None	88%[b]	All Adults	All Adults	6	28	Q, L, PCR37	2016-2018
	Latin America	High income	Non-Metropolitan Regions, Chile	2+1	None	97.90%	None	All Adults	50	240	Q	2017
	Latin America	Upper middle income	Brazil	2+1	None	93%[a]	High Risk	High Risk	613	950	Q, L, PCR70	2015-2018
	Latin America	Upper middle income	Colombia (SIREVA)	2+1	None	92.5%[b]	None	High Risk	281	273	PCR	2017-2018
	Latin America	Upper middle income	Ecuador (IB-VPD)	3+0	None	84.5%[b]	None	None	18	22	PCR	2016-2018
			Ecuador (SIREVA)									2018
	Northern Africa & Western Asia	Lower middle income	Grand Casablanca, Morocco	2+1	None	100%	None	None	12	1	Q, L, PCR70	2017-2018
	Sub-Saharan Africa	Lower middle income	Asembo, Kenya	3+0	None	89.80%	None	None	4	2	Q, PCR	2016
	Sub-Saharan Africa	Lower middle income	Kilifi, Kenya	3+0	None	84.30%	None	None	4	0	Q, L, PCR	2017
PCV13	Europe	High income	Catalonia, Spain	2+1	3+ years	90%	High Risk	All Adults	76	633	Q, PCR37	2016-2018
	Europe	High income	Denmark	2+1	1-2 years	94%[a]	All Adults	All Adults	60	1920	Q, L, PCR70	2014-2019
	Europe	High income	England, UK	2+1	3+ years	94%	None	All Adults	1479	16173	Q, L	2014-2018
	Europe	High income	France	2+1	3+ years	93%	High Risk	High Risk	935	1720	Q, L, PCR70	2013-2017
	Europe	High income	Germany	2+1	3+ years	84%[b]	High Risk	All Adults	675	7725	Q, PCR70	2016-2017
	Europe	High income	Ireland	2+1	1-2 years	89.90%	High Risk	All Adults	52	537	Q, L, PCR	2014-2018
	Europe	High income	Italy	2+1	3+ years	87.7%[a]	All Adults	All Adults	198	2087	Q, L	2014-2018
	Europe	High income	Madrid, Spain	2+1	3+ years	99.30%	All Adults	All Adults	243	1132	Q, L, PCR76	2017-2018
	Europe	High income	Navarra, Spain	2+1	3+ years	93%	High Risk	All Adults	18	99	Q, PCR76	2015-2017
	Europe	High income	Norway	2+1	3+ years	94%	High Risk	All Adults	58	456	Q, NGS	2014-2018
	Europe	High income	Scotland, UK	2+1	3+ years	96.80%	High Risk	All Adults	118	1295	L	2015-2017
	Europe	High income	Switzerland	2+1	3+ years	80.5%[a]	High Risk	None	62	715	Q	2015
	Europe	High income	Belgium	2+1	3+ years	92.9%[a]	N/A	N/A	139	0	Q	2014-2017
	North America	High income	ABCs, USA	3+1	3+ years	88.30%	All Adults	All Adults	606	4120	Q, PCR70, WGS	2014-2017
	North America	High income	Alaska, USA	3+1	3+ years	83.50%	All Adults	All Adults	38	139	Q, L, PCR70	2014-2017

North America	High income	Alberta, Canada	2+1	3+ years	88.1%[a]	High Risk	All Adults	46	171	Q	2014-2017
North America	High income	California, USA	3+1	3+ years	96.60%	All Adults	All Adults	32	301	Q	2015-2018
North America	High income	Ontario, Canada	2+1	3+ years	74.4%[a]	High Risk	All Adults	83	406	Q	2015-2017
North America	High income	Quebec-Nunavik, Canada	3+1	3+ years	98.30%	High Risk	All Adults	9	6	Q	2015-2017
North America	High income	Quebec (excluding Nunavik), Canada	2+1	3+ years	98.30%	High Risk	All Adults	153	626	Q	2014-2018
North America	High income	Southwest, USA (Indigenous)	3+1	3+ years	79%	All Adults	All Adults	29	166	Q	2014-2017
North America	High income	Massachusetts, USA	3+1	3+ years	94.10%	All Adults	All Adults	79	0	Q	2014-2017
North America	High income	Utah, USA	3+1	3+ years	87.10%	All Adults	All Adults	59	0	Q	2017
Latin America	High income	Panama (SIREVA)	2+1	1-2 years	97%[b]	All Adults	All Adults	14	23	Q, PCR37	2016-2017
Latin America	High income	Uruguay (SIREVA)	2+1	1-2 years	94%[b]	None	All Adults	62	220	Q, PCR37	2017-2018
Latin America	Upper middle income	Argentina	2+1	None	82%[b]	All Adults	All Adults	275	69	Q, PCR37, PCR70	2017-2018
Latin America	Upper middle income	Costa Rica	2+1	None	96.50%	High Risk	High Risk	17	51	Q	2017
Latin America	Upper middle income	Mexico (SIREVA)	2+1	3+ years	92%[b]	N/A	N/A	7	0	Q, PCR37, PCR70	2017
Latin America	Lower middle income	Honduras (SIREVA)	3+0	None	89%[b]	None	None	3	1	Q	2017
Latin America	Lower middle income	El Salvador (IB-VPD)	2+1	1-2 years	87%[b]	N/A	N/A	1	0	Q	2017
Latin America	Lower middle income	Nicaragua (IB-VPD)	3+0	None	98%[b]	N/A	N/A	3	0	Q, PCR37	2016-2017
Northern Africa & Western Asia	High income	Israel	2+1	1-2 years	94.30%	All Adults	All Adults	278	702	Q	2016-2018
Sub-Saharan Africa	Upper middle income	South Africa	2+1	1-2 years	73%[a]	All Adults	All Adults	595	754	Q, PCR38	2017
Sub-Saharan Africa	Lower middle income	Cameroon (IB-VPD)	3+0	None	72%[b]	N/A	N/A	3	0	PCR	2017-2018
Sub-Saharan Africa	Low income	Blantyre District, Malawi	3+0	None	95%	None	None	25	5	L, PCR37	2017
Sub-Saharan Africa	Low income	Basse, The Gambia	3+0	1-2 years	80%	N/A	N/A	7	0	L, PCR21	2017
Sub-Saharan Africa	Low income	Benin (IB-VPD)	3+0	None	73%[b]	N/A	N/A	2	0	PCR	2016-2019
Asia	High income	Hong Kong	3+1	1-2 years	97.90%	All Adults	All Adults	106	210	PCR35	2017-2019
Asia	High income	Singapore	2+1	1-2 years	88.30%	All Adults	All Adults	15	141	Q	2018
Asia	High income	Japan	3+1	3+ years	98%[b]	N/A	N/A	96	0	Q, PCR70	2015-2017
Oceania	High income	Australia (Non-Indigenous)	3+0	3+ years	93.80%	All Adults	All Adults	546	1171	Q, PCR	2016-2017
Oceania	High income	Northern Territory, Australia	3+1	3+ years	92.40%	N/A	N/A	16	0	Q, PCR	2016-2017

[1]PCV product and infant schedule that was used in the analysis (which met the mature definition for PCV10 or PCV13). Some sites may have used more than one product or schedule over time and may currently use a different one from that listed here. PCV13 is Pfizer's Prevnar13/Prevenar13; PCV10 is GSK's Synflorix.
[2]UN Region.

[3]World Bank income level.
[4]Number of years of PCV7 use prior to PCV10 or PCV13 introduction.
[5]Median infant PCV coverage in the years eligible for the analysis (met the mature definition for PCV10 or PCV13). Primary series coverage contributed by site, unless otherwise noted.
[6]An adult pneumococcal vaccination program was defined as a PCV13 or PPV23 recommendation for all adults of a certain age or for high-risk populations over 5 years of age during the years eligible for the analysis (met the mature definition for PCV10 or PCV13). Some sites may currently have a different program from the one listed here. N/A (not applicable) shown for sites that did not contribute cases among adults to the analyses.
[7]Number of serotyped IPD cases for each age group during the years eligible for the analysis (met the mature definition for PCV10 or PCV13).
[8]Where available, the number following "PCR" indicates the number of serotypes able to be identified by PCR: PCR37 = real-time polymerase chain reaction (21 assays/37 serotypes): serotypes detected vary by regional scheme; PCR70 = conventional multiplex polymerase chain reaction (41 assays/70 serotypes); PCR35 = conventional multiplex polymerase chain reaction (35 serotypes); PCR38 = real-time polymerase chain reaction (serotypes detected by Africa scheme plus serotype 8); Q = Quellung reaction; L = Latex agglutination; WGS = Whole genome sequencing; NGS = Next generation sequencing; CST = Capsular sequence typing.
[9]Years included in the analysis for either children or adults.
[a] Booster dose coverage.
[b] WUENIC coverage (https://immunizationdata.who.int/pages/coverage/pcv.html).

Table S3. Percentage of IPD cases in the mature PCV10/13 period due to serotypes included in current and upcoming products, corresponding to Figure 2.

Current infant product	Serotype category	Children <5 years	Children 5–17 years	Adults 18–49 years	Adults ≥50 years
**PCV10 sites**	**PCV10**	10.0 (6.3, 12.9)	20.6 (14.1, 29.0)	20.3 (17.3, 23.4)	15.4 (13.4, 19.3)
	**PCV13**	52.1 (49.2, 65.4)	56.5 (47.3, 64.5)	54.9 (46.5, 61.3)	45.6 (40.0, 50.0)
	**PCV15**	56.3 (53.9, 66.9)	56.5 (46.0, 64.2)	59.9 (49.2, 66.7)	50.9 (46.7, 56.6)
	**PCV20**	65.6 (61.4, 74.9)	65.7 (57.0, 75.3)	74.7 (66.0, 80.2)	65.6 (60.4, 71.5)
	**PCV24**	67.7 (63.7, 75.7)	68.0 (59.8, 77.4)	78.4 (70.8, 84.8)	72.1 (67.2, 78.2)
	**PCV25**	83.6 (81.9, 88.5)	77.3 (65.2, 82.5)	83.7 (77.4, 86.3)	80.6 (76.0, 85.8)
	**PPV23**	65.4 (60.4, 71.5)	64.8 (58.4, 74.1)	76.7 (68.0, 83.3)	70.6 (65.5, 77.4)
	**PCV21**	82.0 (77.5, 87.0)	69.7 (53.0, 78.9)	73.8 (66.8, 78.5)	77.5 (68.0, 82.9)
	**PCV21 or PCV13**	91.4 (89.8, 93.6)	85.9 (73.0, 92.7)	92.3 (86.0, 95.2)	90.4 (85.2, 94.0)
	**PCV21-non-PCV13**	38.1 (29.2, 40.9)	35.3 (27.6, 41.5)	38.5 (30.1, 48.1)	46.4 (40.4, 51.9)
**PCV13 sites**	**PCV10**	7.7 (6.1, 9.0)	15.8 (12.8, 19.0)	13.9 (12.2, 16.3)	8.8 (7.9, 11.3)
	**PCV13**	26.4 (21.3, 30.0)	34.2 (28.1, 41.8)	31.5 (28.8, 37.2)	29.5 (27.5, 33.0)
	**PCV15**	36.0 (32.0, 39.9)	39.4 (32.8, 45.7)	37.9 (34.9, 44.4)	39.1 (37.5, 43.0)
	**PCV20**	62.4 (59.5, 65.0)	61.9 (55.2, 65.8)	69.0 (67.1, 72.3)	62.5 (61.0, 64.2)
	**PCV24**	66.2 (63.5, 68.4)	68.4 (62.9, 72.5)	79.0 (76.8, 81.2)	71.4 (69.7, 73.4)
	**PCV25**	76.5 (74.6, 78.7)	74.2 (71.5, 76.4)	80.6 (78.8, 82.9)	76.9 (75.6, 78.6)
	**PPV23**	65.7 (63.0, 67.9)	68.3 (63.2, 72.1)	78.6 (76.3, 81.1)	70.9 (69.2, 73.0)
	**PCV21**	79.3 (77.6, 81.5)	72.6 (65.7, 78.5)	80.3 (75.6, 82.8)	83.3 (79.6, 85.3)
	**PCV21 or PCV13**	86.0 (84.6, 87.7)	86.5 (82.5, 89.8)	91.1 (89.0, 92.9)	90.7 (89.5, 92.2)
	**PCV21-non-PCV13**	60.6 (56.9, 65.2)	53.9 (47.4, 61.1)	61.1 (54.5, 64.2)	61.7 (56.9, 65.2)

Columns display percents with 95% confidence intervals.

Table S4. Serotype-specific distribution of IPD in the mature PCV10/13 period, corresponding to Figure 3.

Vaccine-type	Serotype	Children <5 years		Individuals 5–49 years		Adults ≥50 years	
		PCV10 sites	PCV13 sites	PCV10 sites	PCV13 sites	PCV10 sites	PCV13 sites
PCV7	4	0.4 (0.0, 1.1)	0.1 (0.0, 0.3)	3.8 (2.0, 5.7)	2.7 (1.5, 3.9)	2.9 (1.2, 4.5)	1.2 (0.8, 1.6)
	6B	0.4 (0.0, 1.2)	0.4 (0.2, 0.7)	1.3 (0.2, 2.4)	0.6 (0.3, 0.8)	1.2 (0.1, 2.4)	0.6 (0.4, 0.8)
	9V	0.7 (0.0, 1.4)	0.2 (0.0, 0.4)	1.5 (0.4, 2.5)	0.8 (0.4, 1.2)	1.5 (0.7, 2.3)	0.6 (0.4, 0.9)
	14	4.4 (0.5, 8.2)	1.6 (0.4, 2.7)	2.6 (0.7, 4.5)	1.6 (0.6, 2.6)	2.0 (0.7, 3.3)	1.4 (0.7, 2.2)
	18C	0.9 (0.1, 1.6)	0.4 (0.1, 0.6)	1.0 (0.1, 2.0)	0.8 (0.5, 1.1)	0.8 (0.3, 1.4)	0.4 (0.2, 0.5)
	19F	1.0 (0.0, 2.7)	3.9 (2.5, 5.4)	1.7 (0.3, 3.1)	3.6 (2.4, 4.8)	2.0 (0.6, 3.5)	2.3 (1.6, 2.9)
	23F	1.1 (0.5, 1.8)	0.6 (0.4, 0.8)	0.9 (0.6, 1.2)	0.7 (0.5, 0.8)	0.7 (0.4, 1.0)	0.5 (0.4, 0.6)
PCV10x	1	1.6 (0.0, 3.8)	1.0 (0.5, 1.6)	3.3 (0.2, 6.3)	1.3 (0.7, 1.9)	0.6 (0.0, 1.3)	0.3 (0.2, 0.5)
	5	0.0 (0.0, 0.0)	0.2 (0.0, 0.5)	0.7 (0.0, 1.6)	0.2 (0.0, 0.4)	0.1 (0.0, 0.3)	0.1 (0.0, 0.1)
	7F*	1.3 (0.4, 2.2)	1.0 (0.5, 1.4)	5.1 (3.2, 6.9)	3.3 (2.5, 4.2)	2.9 (1.6, 4.3)	1.8 (1.4, 2.2)
PCV13x	3*	8.4 (5.8, 11.0)	9.6 (6.8, 12.4)	12.9 (7.7, 18.1)	10.7 (8.3, 13.2)	14.3 (11.3, 17.3)	14.5 (12.4, 16.7)
	6A*	2.7 (0.7, 4.7)	0.4 (0.1, 0.7)	2.1 (0.5, 3.7)	0.6 (0.2, 0.9)	1.6 (0.6, 2.5)	0.6 (0.3, 0.9)
	19A*	30.6 (18.2, 43.1)	6.5 (5.1, 8.0)	17.7 (2.0, 23.4)	5.7 (4.5, 6.9)	14.8 (11.9, 17.8)	5.2 (4.3, 6.1)
PCV15x	22F*	2.6 (0.7, 4.4)	5.3 (3.9, 6.7)	3.2 (0.8, 5.7)	5.3 (4.1, 6.4)	5.5 (2.6, 8.4)	7.8 (6.5, 9.2)
	33F*	1.2 (0.0, 3.1)	4.3 (2.8, 5.9)	0.5 (0.0, 1.0)	2.1 (1.4, 2.9)	0.8 (0.0, 1.7)	2.0 (1.4, 2.6)
PCV20x	8*	1.7 (0.1, 3.3)	3.5 (2.3, 4.8)	5.0 (0.0, 10.1)	13.4 (9.4, 17.4)	5.5 (0.3, 10.6)	9.3 (6.1, 12.4)
	10A*	1.4 (0.8, 2.0)	5.5 (4.1, 6.9)	1.8 (0.9, 2.7)	2.5 (1.7, 3.2)	1.8 (0.7, 2.9)	2.8 (2.2, 3.4)
	11A*	1.9 (0.4, 3.3)	2.4 (1.7, 3.0)	2.8 (0.5, 5.1)	2.4 (1.9, 3.0)	3.0 (1.4, 4.6)	3.4 (2.7, 4.1)
	12F*	1.1 (0.0, 2.2)	5.8 (3.4, 8.3)	3.4 (0.0, 7.1)	7.2 (5.3, 9.1)	2.4 (0.2, 4.6)	4.7 (3.5, 6.0)
	15BC*	3.4 (2.1, 4.6)	9.5 (8.4, 10.5)	1.6 (0.9, 2.3)	2.5 (2.1, 2.8)	1.9 (1.2, 2.6)	2.5 (2.3, 2.8)
PCV24x	2	0.0 (0.0, 0.0)	0.2 (0.0, 0.5)	0.0 (0.0, 0.0)	0.3 (0.0, 0.6)	0.0 (0.0, 0.0)	0.1 (0.0, 0.2)
	9N*	1.1 (0.0, 2.4)	2.1 (1.4, 2.8)	2.6 (1.2, 4.0)	5.7 (4.5, 6.9)	4.3 (2.6, 6.0)	5.5 (4.6, 6.4)
	17F*	0.4 (0.0, 1.4)	0.7 (0.4, 1.1)	0.6 (0.0, 1.6)	1.4 (0.6, 2.2)	0.9 (0.2, 1.6)	1.6 (1.2, 2.1)
	20*	0.8 (0.0, 1.7)	1.0 (0.7, 1.2)	1.1 (0.1, 2.1)	2.1 (1.3, 2.8)	1.2 (0.5, 1.9)	2.0 (1.5, 2.5)
PCV25x	15A*	2.8 (0.8, 4.9)	4.0 (2.9, 5.0)	1.8 (0.4, 3.2)	2.6 (2.1, 3.2)	2.7 (1.6, 3.9)	4.3 (3.6, 5.1)
	16F*	0.7 (0.0, 1.6)	1.8 (0.9, 2.6)	1.1 (0.3, 1.9)	1.6 (0.9, 2.2)	1.6 (0.5, 2.8)	2.3 (1.6, 3.1)
	24F*	4.6 (1.8, 7.4)	4.1 (1.2, 7.0)	0.9 (0.0, 1.8)	1.1 (0.4, 1.8)	0.9 (0.6, 1.2)	1.2 (0.5, 1.9)
	35B*	1.4 (0.0, 2.9)	2.3 (1.3, 3.3)	1.3 (0.4, 2.2)	1.3 (0.8, 1.8)	1.6 (1.1, 2.2)	1.9 (1.3, 2.5)
Other	6C	6.3 (4.1, 8.5)	0.8 (0.6, 0.9)	4.7 (2.5, 6.9)	1.7 (1.4, 2.0)	6.0 (4.7, 7.4)	2.5 (2.2, 2.9)
	7C	0.3 (0.0, 0.6)	0.8 (0.3, 1.2)	0.9 (0.0, 1.9)	0.6 (0.3, 1.0)	0.4 (0.0, 0.9)	0.7 (0.4, 1.1)
	13	0.9 (0.1, 1.8)	0.4 (0.0, 0.8)	0.7 (0.0, 1.4)	0.3 (0.1, 0.5)	0.5 (0.0, 1.2)	0.4 (0.1, 0.6)
	18A	0.2 (0.0, 0.5)	0.1 (0.0, 0.2)	0.6 (0.0, 1.4)	0.4 (0.1, 0.6)	0.3 (0.0, 1.0)	0.2 (0.1, 0.3)
	21	0.2 (0.0, 0.7)	1.5 (1.0, 2.0)	0.3 (0.0, 0.5)	0.5 (0.3, 0.7)	0.1 (0.0, 0.3)	0.4 (0.2, 0.5)
	23A*	2.1 (0.0, 4.2)	1.6 (1.2, 2.1)	2.5 (1.2, 3.7)	2.5 (1.9, 3.1)	3.5 (1.8, 5.1)	3.4 (2.7, 4.2)
	23B*	3.2 (1.1, 5.3)	5.3 (3.5, 7.1)	2.0 (1.2, 2.9)	2.9 (2.2, 3.6)	2.6 (2.0, 3.2)	2.5 (1.9, 3.0)
	25A	1.3 (0.2, 2.4)	0.3 (0.1, 0.5)	0.3 (0.0, 0.6)	0.0 (0.0, 0.1)	0.2 (0.0, 0.6)	0.1 (0.0, 0.2)
	28A	0.7 (0.2, 1.2)	0.3 (0.1, 0.4)	0.4 (0.1, 0.8)	0.2 (0.1, 0.3)	0.4 (0.0, 0.9)	0.2 (0.1, 0.3)
	31*	0.5 (0.0, 1.3)	0.6 (0.3, 0.9)	0.5 (0.0, 1.0)	0.9 (0.6, 1.3)	0.6 (0.1, 1.0)	1.9 (1.5, 2.3)
	34	0.6 (0.2, 0.9)	0.4 (0.3, 0.5)	0.6 (0.3, 1.0)	0.6 (0.5, 0.7)	0.7 (0.4, 1.0)	0.6 (0.6, 0.7)
	35F	0.7 (0.0, 1.9)	1.4 (0.9, 1.8)	0.6 (0.1, 1.0)	1.1 (0.7, 1.5)	1.2 (0.3, 2.1)	1.6 (1.2, 2.0)
	38	0.6 (0.0, 2.0)	2.3 (1.4, 3.2)	0.2 (0.0, 0.8)	0.5 (0.2, 0.7)	0.4 (0.0, 0.8)	1.2 (0.8, 1.6)

Columns display percents with 95% confidence intervals.
*Serotype included in PCV21.

Table S5. Percentage of IPD cases in the mature PCV13 period due to serotypes included in current and upcoming products, stratified by finer age groups. Data at PCV10 sites were insufficient for further age stratification.

Serotype Category	Children <5 years		Adults ≥50 years			
	**<2y**	**2-4y**	**50-64y**	**65-74y**	**75-84y**	**85+y**
**PCV10**	6.8 (5.1, 8.4)	9.3 (6.9, 11.3)	9.7 (8.5, 11.4)	7.8 (6.5, 9.0)	7.5 (6.4, 8.7)	7.6 (6.1, 9.5)
**PCV13**	22.5 (18.3, 26.0)	33.4 (25.5, 37.1)	27.8 (25.4, 29.6)	26.9 (25.0, 29.3)	27.0 (25.5, 29.8)	27.2 (23.2, 29.4)
**PCV15**	33.1 (29.4, 35.7)	40.7 (31.7, 44.1)	37.9 (35.3, 39.7)	37.0 (35.0, 39.1)	37.8 (34.6, 39.4)	37.9 (35.4, 40.8)
**PCV20**	61.2 (59.7, 64.0)	64.9 (60.6, 68.5)	65.8 (63.6, 67.9)	63.4 (61.6, 65.6)	60.8 (58.9, 62.5)	58.1 (54.6, 60.6)
**PCV24**	65.9 (63.9, 68.1)	67.7 (63.9, 71.6)	76.2 (74.1, 77.9)	71.9 (70.2, 73.7)	69.0 (66.8, 71.1)	66.4 (63.4, 69.1)
**PCV25**	75.9 (73.2, 77.6)	77.8 (73.3, 80.6)	79.5 (77.7, 81.1)	78.1 (76.8, 79.8)	75.8 (72.8, 77.7)	75.3 (73.4, 77.1)
**PPV23**	65.6 (63.1, 67.3)	67.1 (63.4, 71.8)	75.8 (73.8, 77.5)	71.5 (69.6, 73.2)	68.5 (66.2, 70.7)	65.8 (63.1, 68.6)
**PCV21**	79.0 (76.2, 80.9)	81.4 (76.8, 83.7)	85.0 (81.8, 86.5)	85.4 (83.5, 87.8)	84.3 (82.1, 86.2)	83.8 (81.9, 85.8)
**PCV21 or PCV13**	84.9 (82.6, 86.3)	89.5 (86.5, 90.7)	92.4 (90.6, 93.4)	91.9 (90.8, 93.7)	90.7 (89.4, 91.9)	90.4 (89.3, 92.0)
**PCV21-non-PCV13**	64.6 (61.3, 68.5)	58.6 (55.2, 65.6)	65.2 (62.2, 68.2)	65.6 (63.4, 68.8)	64.1 (59.7, 66.0)	63.6 (60.1, 67.3)

Columns display percents with 95% confidence intervals.

Table S6. Serotype-specific distribution of IPD in the mature PCV13 period, stratified by finer age groups (corresponds to Figure S5). Data at PCV10 sites were insufficient for further age stratification.

Vaccine-type	Serotype	Children <5 years		Adults ≥50 years			
		<2y	2–4y	50–64y	65–74y	75–84y	≥85y
PCV7	4	0.1 (0.0, 0.2)	0.2 (0.0, 0.4)	1.8 (1.0, 2.5)	1.0 (0.5, 1.6)	0.5 (0.2, 0.8)	0.4 (0.2, 0.7)
	6B	0.4 (0.1, 0.7)	0.3 (0.0, 0.7)	0.4 (0.2, 0.5)	0.4 (0.2, 0.6)	0.6 (0.2, 0.9)	0.7 (0.3, 1.1)
	9V	0.2 (0.0, 0.3)	0.3 (0.0, 0.7)	0.6 (0.3, 1.0)	0.5 (0.2, 0.8)	0.4 (0.2, 0.7)	0.7 (0.2, 1.1)
	14	1.4 (0.2, 2.6)	1.9 (0.3, 3.5)	1.0 (0.4, 1.7)	1.2 (0.6, 1.8)	1.2 (0.4, 2.0)	1.6 (0.5, 2.8)
	18C	0.2 (0.0, 0.5)	0.5 (0.2, 0.9)	0.5 (0.2, 0.7)	0.3 (0.1, 0.5)	0.3 (0.1, 0.5)	0.3 (0.1, 0.5)
	19F	3.6 (2.2, 5.0)	3.8 (1.8, 5.7)	2.3 (1.5, 3.1)	2.3 (1.4, 3.2)	2.2 (1.4, 3.1)	2.1 (1.4, 2.9)
	23F	0.4 (0.3, 0.6)	0.5 (0.3, 0.7)	0.5 (0.3, 0.6)	0.4 (0.3, 0.5)	0.5 (0.4, 0.6)	0.2 (0.2, 0.3)
PCV10x	1	0.5 (0.1, 0.8)	1.9 (0.9, 2.9)	0.4 (0.1, 0.7)	0.2 (0.1, 0.3)	0.3 (0.1, 0.4)	0.1 (0.0, 0.1)
	5	0.1 (0.0, 0.3)	0.2 (0.0, 0.4)	0.0 (0.0, 0.1)	0.0 (0.0, 0.1)	0.0 (0.0, 0.0)	0.0 (0.0, 0.1)
	7F*	0.8 (0.3, 1.3)	0.5 (0.2, 0.9)	2.0 (1.5, 2.5)	1.2 (0.8, 1.6)	1.3 (1.0, 1.6)	1.0 (0.5, 1.5)
PCV13x	3*	8.0 (6.0, 10.1)	11.9 (7.6, 16.2)	12.2 (10.5, 13.8)	13.5 (11.7, 15.3)	14.2 (12.8, 15.7)	13.3 (11.7, 14.8)
	6A*	0.2 (0.0, 0.4)	0.7 (0.1, 1.3)	0.5 (0.2, 0.8)	0.4 (0.1, 0.7)	0.6 (0.3, 1.0)	0.6 (0.2, 1.0)
	19A*	5.5 (4.2, 6.7)	8.6 (6.4, 10.8)	4.9 (4.0, 5.7)	5.2 (4.1, 6.2)	4.9 (3.8, 6.0)	5.7 (4.3, 7.1)
PCV15x	22F*	5.2 (3.8, 6.7)	5.5 (3.2, 7.8)	8.3 (6.5, 10.1)	8.2 (6.4, 9.9)	8.6 (6.9, 10.2)	8.7 (6.8, 10.5)
	33F*	5.7 (3.2, 8.3)	2.0 (1.2, 2.9)	2.4 (1.8, 2.9)	2.4 (1.7, 3.1)	2.4 (1.6, 3.2)	2.6 (1.8, 3.4)
PCV20x	8*	4.4 (2.5, 6.3)	3.0 (1.8, 4.2)	12.6 (8.0, 17.2)	11.3 (6.6, 16.1)	9.9 (5.7, 14.1)	7.3 (4.3, 10.3)
	10A*	6.8 (5.0, 8.6)	3.5 (2.4, 4.7)	3.1 (2.4, 3.9)	2.9 (2.5, 3.4)	2.4 (1.7, 3.0)	2.5 (1.6, 3.4)
	11A*	2.4 (1.6, 3.2)	2.3 (1.4, 3.1)	3.0 (2.3, 3.7)	3.7 (2.5, 4.9)	3.8 (2.6, 5.0)	3.6 (2.8, 4.4)
	12F*	6.2 (3.7, 8.7)	7.7 (4.5, 10.9)	6.4 (4.6, 8.2)	4.9 (3.7, 6.0)	4.1 (3.0, 5.2)	3.7 (2.5, 4.8)
	15BC*	9.5 (8.4, 10.6)	9.1 (7.6, 10.7)	2.3 (1.9, 2.6)	2.9 (2.5, 3.2)	2.4 (2.1, 2.7)	2.9 (2.5, 3.3)
PCV24x	2	0.2 (0.0, 0.6)	0.1 (0.0, 0.2)	0.1 (0.0, 0.2)	0.0 (0.0, 0.1)	0.1 (0.0, 0.1)	0.0 (0.0, 0.1)
	9N*	2.5 (1.7, 3.3)	1.7 (1.0, 2.4)	6.7 (5.3, 8.0)	5.9 (4.6, 7.2)	5.3 (4.3, 6.4)	5.5 (4.1, 6.9)
	17F*	0.9 (0.4, 1.4)	0.7 (0.2, 1.2)	1.8 (1.2, 2.3)	1.2 (0.8, 1.7)	1.5 (0.9, 2.0)	1.7 (1.0, 2.3)
	20*	1.3 (0.9, 1.7)	0.6 (0.3, 0.8)	2.3 (1.7, 2.9)	1.8 (1.3, 2.2)	1.8 (1.4, 2.1)	1.4 (1.1, 1.7)
PCV25x	15A*	3.9 (2.9, 5.0)	4.0 (2.4, 5.7)	3.3 (2.5, 4.1)	4.3 (3.2, 5.3)	4.5 (3.5, 5.6)	5.5 (3.8, 7.2)
	16F*	1.9 (0.9, 2.8)	1.6 (0.3, 2.8)	1.9 (1.3, 2.5)	2.5 (1.5, 3.4)	2.8 (1.8, 3.7)	2.1 (1.2, 3.0)
	24F*	4.8 (1.3, 8.2)	4.4 (1.1, 7.7)	1.2 (0.3, 2.0)	1.4 (0.5, 2.3)	1.7 (0.8, 2.6)	1.9 (0.9, 2.8)
	35B*	2.5 (1.2, 3.8)	1.4 (0.5, 2.2)	1.6 (1.0, 2.2)	2.0 (1.3, 2.8)	2.2 (1.3, 3.1)	2.5 (1.5, 3.6)
Other	6C	0.4 (0.3, 0.5)	1.0 (0.7, 1.2)	2.2 (1.9, 2.5)	2.2 (1.8, 2.6)	2.4 (2.0, 2.8)	3.1 (2.6, 3.5)
	7C	0.9 (0.3, 1.4)	0.5 (0.0, 0.9)	0.6 (0.3, 1.0)	0.6 (0.3, 1.0)	0.9 (0.4, 1.4)	0.9 (0.5, 1.3)
	13	0.4 (0.0, 0.8)	0.4 (0.0, 0.8)	0.3 (0.0, 0.5)	0.4 (0.1, 0.7)	0.2 (0.1, 0.4)	0.3 (0.0, 0.5)
	18A	0.2 (0.0, 0.4)	0.0 (0.0, 0.0)	0.2 (0.1, 0.4)	0.1 (0.0, 0.1)	0.2 (0.0, 0.4)	0.1 (0.0, 0.3)
	21	1.7 (1.0, 2.4)	1.3 (0.7, 1.9)	0.3 (0.1, 0.4)	0.4 (0.2, 0.6)	0.4 (0.2, 0.6)	0.2 (0.0, 0.3)
	23A*	1.3 (0.8, 1.8)	2.0 (1.1, 3.0)	2.7 (1.9, 3.5)	3.4 (2.6, 4.1)	3.7 (2.5, 4.9)	4.2 (3.0, 5.3)
	23B*	4.2 (2.6, 5.8)	7.2 (4.5, 9.9)	2.5 (1.9, 3.1)	2.6 (1.9, 3.3)	2.9 (2.2, 3.5)	2.1 (1.7, 2.6)
	25A	0.3 (0.0, 0.5)	0.3 (0.0, 0.6)	0.1 (0.0, 0.2)	0.1 (0.0, 0.2)	0.2 (0.0, 0.4)	0.1 (0.0, 0.3)
	28A	0.4 (0.1, 0.6)	0.1 (0.0, 0.2)	0.2 (0.1, 0.4)	0.1 (0.0, 0.3)	0.1 (0.0, 0.2)	0.1 (0.0, 0.2)
	31*	0.5 (0.2, 0.8)	0.8 (0.3, 1.4)	1.4 (0.9, 1.8)	1.8 (1.3, 2.3)	2.0 (1.5, 2.5)	3.0 (2.2, 3.9)
	34	0.4 (0.3, 0.5)	0.3 (0.2, 0.5)	0.6 (0.5, 0.6)	0.5 (0.4, 0.5)	0.6 (0.5, 0.7)	0.6 (0.5, 0.7)
	35F	1.1 (0.5, 1.6)	1.8 (1.0, 2.6)	1.6 (1.2, 2.1)	1.8 (1.3, 2.3)	2.1 (1.6, 2.7)	1.8 (1.2, 2.3)
	38	2.6 (1.4, 3.7)	1.9 (0.8, 3.0)	0.8 (0.5, 1.1)	1.4 (0.9, 1.9)	1.4 (0.8, 2.0)	2.0 (1.3, 2.7)

Columns display percents with 95% confidence intervals.
*Serotype included in PCV21.

Table S7. Percent of IPD due to serotypes included in current and upcoming PCVs, by region, corresponding to data shown in Figure 4.

Age	Product	Vaccine	Region	Number of sites[a]	Median % coverage	IQR (%)[b]
**Children <5 years**	PCV10 Sites	PCV10	Europe	3	5.1	2.5, 9.4
			Latin America	4	4.9	3.5, 6.4
		PCV13	Europe	3	50.0	44.8, 58.1
			Latin America	4	56.8	46.9, 68.0
		PCV15	Latin America	4	58.6	51.8, 68.6
		PCV20	Latin America	4	67.3	64.2, 72.8
		PCV24	Latin America	4	70.1	67.0, 74.7
		PCV25	Latin America	4	85.2	84.2, 86.7
		PPV23	Latin America	4	66.6	64.9, 69.5
		PCV21	Latin America	4	87.8	85.2, 88.3
		PCV21 or PCV13	Latin America	4	92.1	90.9, 92.9
	PCV13 Sites	PCV10	Europe	13	5.6	4.3, 5.8
			North America	10	5.3	2.7, 6.9
			Latin America	5	11.8	0.0, 18.0
			Sub-Saharan Africa	3	14.3	13.4, 17.1
		PCV13	Europe	13	18.6	13.9, 20.2
			North America	10	19.8	16.5, 22.9
			Latin America	5	47.5	47.1, 50.0
			Sub-Saharan Africa	3	23.1	21.5, 25.8
		PCV15	Europe	13	27.8	23.1, 36.8
			North America	10	39.0	32.1, 43.7
			Latin America	5	50.0	47.1, 52.5
		PCV20	Europe	13	63.9	55.8, 64.7
			North America	10	60.4	58.9, 67.3
			Latin America	5	76.9	76.5, 77.0
		PCV24	Europe	13	66.2	58.3, 68.9
			North America	10	65.4	63.0, 72.0
			Latin America	5	78.7	76.9, 82.4
		PCV25	Europe	12	77.4	74.7, 79.9
			North America	10	74.0	69.8, 81.8
			Latin America	4	75.0	70.8, 80.3
		PPV23	Europe	13	65.8	58.3, 68.9
			North America	10	65.3	62.7, 71.8
			Latin America	5	78.7	61.5, 82.4
		PCV21	Europe	12	82.3	80.0, 88.2
			North America	10	82.4	81.4, 85.6
			Latin America	4	76.4	70.1, 80.4
		PCV21 or PCV13	Europe	12	89.0	86.1, 91.8
			North America	10	87.9	84.8, 88.8
			Latin America	4	82.1	78.1, 87.3
Adults ≥50 years	PCV10 Sites	PCV10	Europe	5	16.7	12.8, 17.9
			Latin America	4	16.4	13.4, 22.7
		PCV13	Europe	5	40.5	40.0, 54.0
			Latin America	4	47.0	44.9, 52.6
		PCV15	Europe	4	48.6	45.1, 55.6
			Latin America	4	49.5	47.9, 55.8
		PCV20	Europe	4	72.3	67.0, 75.5
			Latin America	4	58.4	56.2, 61.4
		PCV24	Europe	4	78.8	77.3, 80.7
			Latin America	4	64.2	61.5, 66.9
		PCV25	Europe	4	86.5	85.3, 87.5
			Latin America	4	74.0	68.9, 77.1
		PPV23	Europe	4	77.8	75.7, 80.5
			Latin America	4	61.7	57.3, 66.1
		PCV21	Europe	4	82.3	78.5, 86.6
			Latin America	4	74.4	67.0, 76.1
		PCV21 or PCV13	Europe	4	95.2	92.5, 96.6
			Latin America	4	88.4	82.4, 89.8
	PCV13 Sites	PCV10	Europe	12	6.3	4.9, 10.1
			North America	8	6.2	3.9, 9.2
			Latin America	4	15.0	9.5, 20.7
		PCV13	Europe	12	25.5	20.8, 31.0

			North America	8	26.2	22.9, 30.3
			Latin America	4	39.2	37.7, 40.3
		PCV15	Europe	12	37.2	32.1, 41.4
			North America	8	40.1	36.3, 45.5
			Latin America	4	42.8	41.4, 44.9
		PCV20	Europe	12	65.8	64.6, 67.2
			North America	8	57.2	54.4, 61.4
			Latin America	4	59.6	55.7, 64.3
		PCV24	Europe	12	75.1	72.2, 77.2
			North America	8	68.2	64.9, 71.6
			Latin America	4	65.4	60.1, 70.2
		PCV25	Europe	11	80.4	77.9, 82.0
			North America	8	73.2	71.4, 76.0
			Latin America	3	76.8	73.2, 76.8
		PPV23	Europe	12	75.0	71.9, 76.7
			North America	8	67.6	64.8, 71.3
			Latin America	4	64.5	58.6, 69.8
		PCV21	Europe	11	88.5	81.6, 91.0
			North America	8	87.3	83.2, 90.7
			Latin America	3	69.6	63.0, 74.3
		PCV21 or PCV13	Europe	11	93.5	92.6, 94.1
			North America	8	92.0	91.2, 93.6
			Latin America	3	84.1	76.8, 85.2

[a] Sites with <5 cases are excluded, and age/product/region strata with <3 sites are not shown as it is insufficient data to compute an IQR.

[b] IQR should be interpreted in the context of the number of sites in each stratum. Stratum with few sites may not be representative of the entire region.

Table S8 (A-B). Summary of sites included in analysis by product and age group, showing additional stratifications to those in Table 1.

A) Children <5 years

Infant PCV Product	Category	Value	Number (col. %) of sites[1]	Number (row %) of sites by case count group[2]				Total cases[3]	Median PCV coverage (IQR)[4]	Number (col. %) used PCV7[5]	Median years of PCV7 use (IQR)[6]
				<20 cases	20-49 cases	50-99 cases	100+ cases				
PCV10	Total PCV10-using sites		12 (100%)	7 (58.3%)	0 (0%)	3 (25%)	2 (16.7%)	1109	93 (89, 94)	2 (16.7%)	0 (0, 0)
	Region	Europe	5 (41.7%)	3 (60%)	0 (0%)	2 (40%)	0 (0%)	127	93 (89, 94)	2 (40%)	0 (0, 2)
		North America	0 (0%)	--	--	--	--	--	--	--	--
		Latin America	4 (33.3%)	1 (25%)	0 (0%)	1 (25%)	2 (50%)	962	93 (90, 94)	0 (0%)	0 (0, 0)
		Sub-Saharan Africa	2 (16.7%)	2 (100%)	0 (0%)	0 (0%)	0 (0%)	8	87 (86, 88)	0 (0%)	0 (0, 0)
		Northern Africa & Western Asia	1 (8.3%)	1 (100%)	0 (0%)	0 (0%)	0 (0%)	12	100	0 (0%)	0
		Asia	0 (0%)	--	--	--	--	--	--	--	--
		Oceania	0 (0%)	--	--	--	--	--	--	--	--
	World Bank Income Level	High income	5 (41.7%)	2 (40%)	0 (0%)	3 (60%)	0 (0%)	171	94 (93, 96)	2 (40%)	0 (0, 2)
		Upper middle income	4 (33.3%)	2 (50%)	0 (0%)	0 (0%)	2 (50%)	918	90 (87, 93)	0 (0%)	0 (0, 0)
		Lower middle income	3 (25%)	3 (100%)	0 (0%)	0 (0%)	0 (0%)	20	90 (87, 95)	0 (0%)	0 (0, 0)
		Low income	0 (0%)	--	--	--	--	--	--	--	--
	Infant schedule	2+1	8 (66.7%)	3 (37.5%)	0 (0%)	3 (37.5%)	2 (25%)	1077	94 (93, 97)	2 (25%)	0 (0, 0)
		3+1	1 (8.3%)	1 (100%)	0 (0%)	0 (0%)	0 (0%)	6	88	0 (0%)	0
		3+0	3 (25%)	3 (100%)	0 (0%)	0 (0%)	0 (0%)	26	84 (84, 87)	0 (0%)	0 (0, 0)
PCV13	Total PCV13-using sites		42 (100%)	13 (31%)	5 (11.9%)	10 (23.8%)	14 (33.3%)	7318	93 (87, 95)	35 (83.3%)	4 (2, 7)
	Region	Europe	13 (31%)	1 (7.7%)	0 (0%)	5 (38.5%)	7 (53.8%)	4113	93 (90, 94)	13 (100%)	4 (3, 5)
		North America	10 (23.8%)	1 (10%)	4 (40%)	3 (30%)	2 (20%)	1134	88 (84, 96)	10 (100%)	10 (8, 10)
		Latin America	8 (19%)	6 (75%)	0 (0%)	1 (12.5%)	1 (12.5%)	382	93 (88, 97)	4 (50%)	0 (0, 1)
		Sub-Saharan Africa	5 (11.9%)	3 (60%)	1 (20%)	0 (0%)	1 (20%)	632	73 (73, 81)	2 (40%)	0 (0, 2)
		Northern Africa & Western Asia	1 (2.4%)	0 (0%)	0 (0%)	0 (0%)	1 (100%)	278	94	1 (100%)	1
		Asia	3 (7.1%)	1 (33.3%)	0 (0%)	1 (33.3%)	1 (33.3%)	217	98 (93, 98)	3 (100%)	3 (2, 3)
		Oceania	2 (4.8%)	1 (50%)	0 (0%)	0 (0%)	1 (50%)	562	93 (93, 93)	2 (100%)	8 (7, 10)
		High income	31 (73.8%)	5 (16.1%)	4 (12.9%)	10 (32.3%)	12 (38.7%)	6380	93 (88, 95)	31 (100%)	5 (3, 9)

	**World Bank Income Level**	**Upper middle income**	4 (9.5%)	2 (50%)	0 (0%)	0 (0%)	2 (50%)	894	87 (80, 93)	2 (50%)	1 (0, 2)
		**Lower middle income**	4 (9.5%)	4 (100%)	0 (0%)	0 (0%)	0 (0%)	10	88 (83, 91)	1 (25%)	0 (0, 0)
		**Low income**	3 (7.1%)	2 (66.7%)	1 (33.3%)	0 (0%)	0 (0%)	34	81 (77, 88)	1 (33.3%)	0 (0, 1)
	**Infant schedule**	**2+1**	25 (59.5%)	6 (24%)	1 (4%)	7 (28%)	11 (44%)	5659	93 (88, 94)	23 (92%)	4 (2, 5)
		**3+1**	10 (23.8%)	2 (20%)	3 (30%)	3 (30%)	2 (20%)	1070	93 (87, 98)	10 (100%)	10 (9, 10)
		**3+0**	7 (16.7%)	5 (71.4%)	1 (14.3%)	0 (0%)	1 (14.3%)	589	89 (77, 94)	2 (28.6%)	0 (0, 1)

B) Adults ≥50 years

Infant PCV Product	Category	Value	Number (col. %) of sites[1]	Number (row %) of sites by case count group[2]				Total cases[3]	Median infant PCV coverage (IQR)[4]	Number (col. %) used infant PCV7[5]	Median years of infant PCV7 use (IQR)[6]
				<20 cases	20-49 cases	50-99 cases	100+ cases				
PCV10	Total PCV10-using sites		11 (100%)	2 (18.2%)	3 (27.3%)	1 (9.1%)	5 (45.5%)	3978	92 (90, 95)	3 (27.3%)	0 (0, 0)
	Region	Europe	5 (45.5%)	0 (0%)	2 (40%)	1 (20%)	2 (40%)	2490	93 (90, 93)	2 (40%)	0 (0, 2)
		North America	0 (0%)	--	--	--	--	--	--	--	--
		Latin America	4 (36.4%)	0 (0%)	1 (25%)	0 (0%)	3 (75%)	1485	91 (89, 93)	1 (25%)	0 (0, 0)
		Sub-Saharan Africa	1 (9.1%)	1 (100%)	0 (0%)	0 (0%)	0 (0%)	2	88 (88, 88)	0 (0%)	0 (0, 0)
		Northern Africa & Western Asia	1 (9.1%)	1 (100%)	0 (0%)	0 (0%)	0 (0%)	1	100	0 (0%)	0
		Asia	0 (0%)	--	--	--	--	--	--	--	--
		Oceania	0 (0%)	--	--	--	--	--	--	--	--
	World Bank Income Level	High income	5 (45.5%)	0 (0%)	1 (20%)	1 (20%)	3 (60%)	2702	93 (93, 96)	2 (40%)	0 (0, 2)
		Upper middle income	4 (36.4%)	0 (0%)	2 (50%)	0 (0%)	2 (50%)	1273	90 (88, 91)	1 (25%)	0 (0, 0)
		Lower middle income	2 (18.2%)	2 (100%)	0 (0%)	0 (0%)	0 (0%)	3	94 (91, 97)	0 (0%)	0 (0, 0)
		Low income	0 (0%)	--	--	--	--	--	--	--	--
	Infant schedule	2+1	8 (72.7%)	1 (12.5%)	1 (12.5%)	1 (12.5%)	5 (62.5%)	3926	93 (92, 97)	2 (25%)	0 (0, 0)
		3+1	1 (9.1%)	0 (0%)	1 (100%)	0 (0%)	0 (0%)	28	90	0 (0%)	0
		3+0	2 (18.2%)	1 (50%)	1 (50%)	0 (0%)	0 (0%)	24	86 (85, 87)	1 (50%)	0 (0, 0)
	Adult Vaccination Program	PCV13 & PPV23	5 (45.5%)	0 (0%)	2 (40%)	0 (0%)	3 (60%)	3390	92 (90, 93)	1 (20%)	0 (0, 0)
		PCV13	0 (0%)	--	--	--	--	--	--	--	--
		PPV23	2 (18.2%)	0 (0%)	0 (0%)	0 (0%)	2 (100%)	513	94 (93, 96)	0 (0%)	0 (0, 0)
		None	4 (36.4%)	2 (50%)	1 (25%)	1 (25%)	0 (0%)	75	90 (87, 95)	2 (50%)	0 (0, 0)
PCV13	Total PCV13-using sites		31 (100%)	3 (9.7%)	1 (3.2%)	3 (9.7%)	24 (77.4%)	43774	90 (86, 95)	27 (87.1%)	4 (2, 7)
	Region	Europe	12 (38.7%)	0 (0%)	0 (0%)	1 (8.3%)	11 (91.7%)	34492	92 (90, 94)	12 (100%)	4 (3, 5)
		North America	8 (25.8%)	1 (12.5%)	0 (0%)	0 (0%)	7 (87.5%)	5935	82 (80, 88)	8 (100%)	9 (8, 10)
		Latin America	5 (16.1%)	1 (20%)	1 (20%)	2 (40%)	1 (20%)	364	94 (89, 97)	2 (40%)	0 (0, 1)

		**Sub-Saharan Africa**	2 (6.5%)	1 (50%)	0 (0%)	0 (0%)	1 (50%)	759	83 (77, 89)	1 (50%)	1 (0, 2)
		**Northern Africa & Western Asia**	1 (3.2%)	0 (0%)	0 (0%)	0 (0%)	1 (100%)	702	94	1 (100%)	1
		**Asia**	2 (6.5%)	0 (0%)	0 (0%)	0 (0%)	2 (100%)	351	93 (91, 96)	2 (100%)	2 (2, 3)
		**Oceania**	1 (3.2%)	0 (0%)	0 (0%)	0 (0%)	1 (100%)	1171	94 (94, 94)	1 (100%)	6 (6, 6)
	**World Bank Income Level**	**High income**	26 (83.9%)	1 (3.8%)	1 (3.8%)	1 (3.8%)	23 (88.5%)	42894	91 (85, 94)	26 (100%)	5 (3, 8)
		**Upper middle income**	3 (9.7%)	0 (0%)	0 (0%)	2 (66.7%)	1 (33.3%)	874	88 (79, 93)	1 (33.3%)	0 (0, 1)
		**Lower middle income**	1 (3.2%)	1 (100%)	0 (0%)	0 (0%)	0 (0%)	1	89 (89, 89)	0 (0%)	0 (0, 0)
		**Low income**	1 (3.2%)	1 (100%)	0 (0%)	0 (0%)	0 (0%)	5	95 (95, 95)	0 (0%)	0 (0, 0)
	**Infant schedule**	**2+1**	22 (71%)	0 (0%)	1 (4.5%)	3 (13.6%)	18 (81.8%)	37655	91 (88, 94)	20 (90.9%)	4 (2, 6)
		**3+1**	6 (19.4%)	1 (16.7%)	0 (0%)	0 (0%)	5 (83.3%)	4942	85 (81, 94)	6 (100%)	10 (9, 10)
		**3+0**	3 (9.7%)	2 (66.7%)	0 (0%)	0 (0%)	1 (33.3%)	1177	94 (92, 94)	1 (33.3%)	0 (0, 3)
	**Adult Vaccination Program**	**PCV13 & PPV23**	26 (83.9%)	1 (3.8%)	1 (3.8%)	3 (11.5%)	21 (80.8%)	26660	90 (85, 95)	24 (92.3%)	4 (2, 8)
		**PCV13**	1 (3.2%)	0 (0%)	0 (0%)	0 (0%)	1 (100%)	715	83	1 (100%)	5
		**PPV23**	2 (6.5%)	0 (0%)	0 (0%)	0 (0%)	2 (100%)	16393	94 (94, 94)	2 (100%)	3 (2, 4)
		**None**	2 (6.5%)	2 (100%)	0 (0%)	0 (0%)	0 (0%)	6	92 (90, 94)	0 (0%)	0 (0, 0)

[1]Total number of sites with eligible data in that age group, and the distribution of sites by category.
[2]Distribution of sites for each category value by case count groups.
[3]Sum of eligible cases across sites in each category value.
[4]The average infant PCV coverage was estimated for each site in the years eligible for the respective analysis, which differed by age group. Here are shown the median and interquartile range (IQR) of those averages by category value.
[5]Number of sites where PCV7 was used in the infant immunization program prior to PCV10/13 introduction.
[6]The mean of the number of years PCV7 was used at each site prior to PCV10/13 introduction.

Number of serotyped IPD cases per site in mature PCV10/13 years for children <5 years and adults ≥50 years by pneumococcal conjugate vaccine (PCV) product used during years included in the analysis, UN region, World Bank income level, and adult pneumococcal vaccination program. UN Regions include Europe, North America, Latin America and Caribbean, Sub-Saharan Africa, Northern Africa and Western Asia, Asia, and Oceania. An adult pneumococcal vaccination program was defined as a PCV13 or PPV23 recommendation for all adults of a certain age or for high risk populations ≥5 years of age. PCV13 is Pfizer's Prevnar13/Prevenar13; PCV10 is GSK's Synflorix.

Supplementary Figures

Figure S1 (A-B). Serotype distribution within the subgroup of PCV13-related serotypes and subgroup of non-PCV13-related serotypes. PCV13-related serotypes include PCV13 serotypes plus 6C. The proportions are among those in the subgroup and sum to 100% within subgroup. For example, among children aged <5 years in PCV10 sites, more than half (52.4%) of PCV13-related IPD was caused by ST19A. Analyses are for IPD cases remaining in mature PCV10/13 settings.

A) Children <5 years

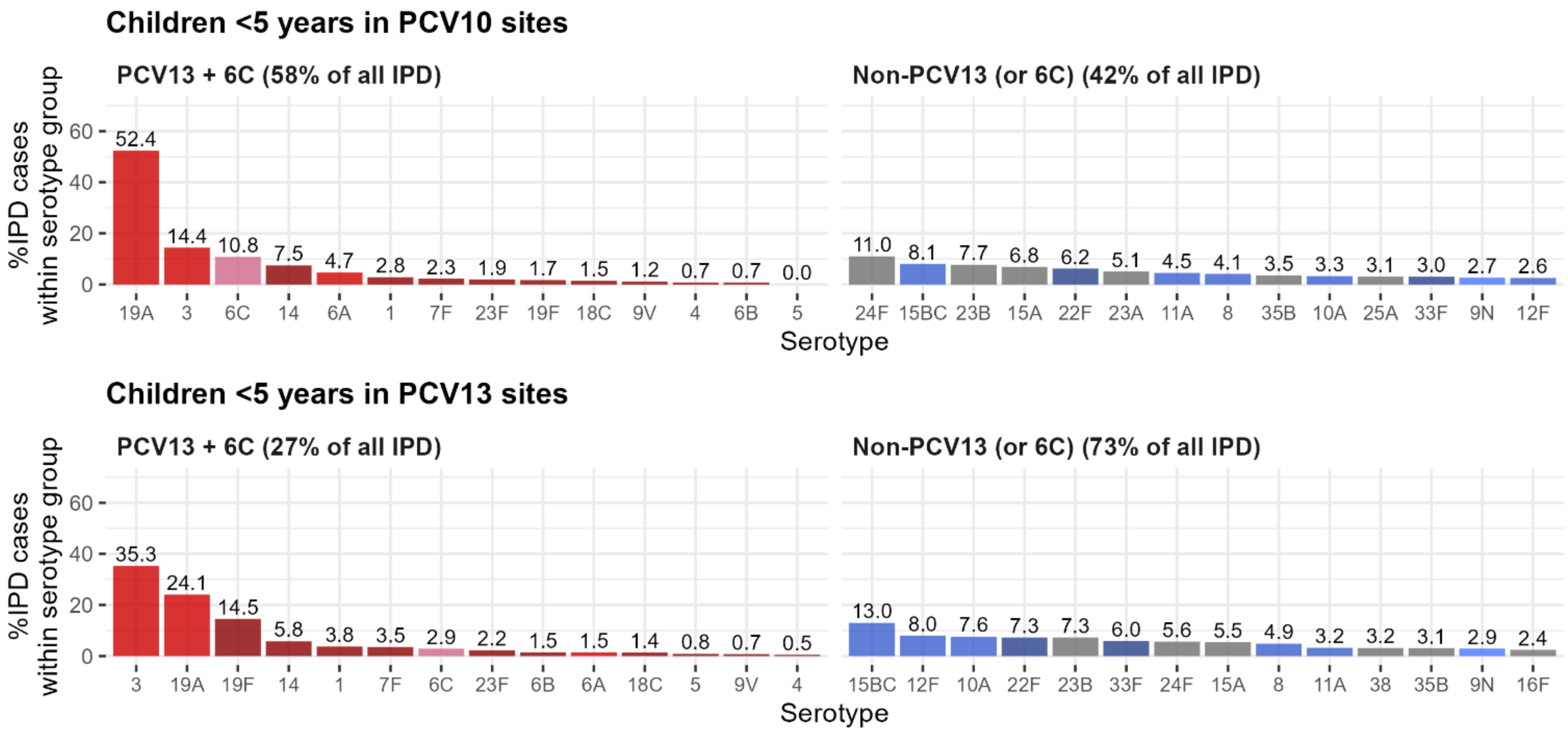

B) Adults ≥50 years

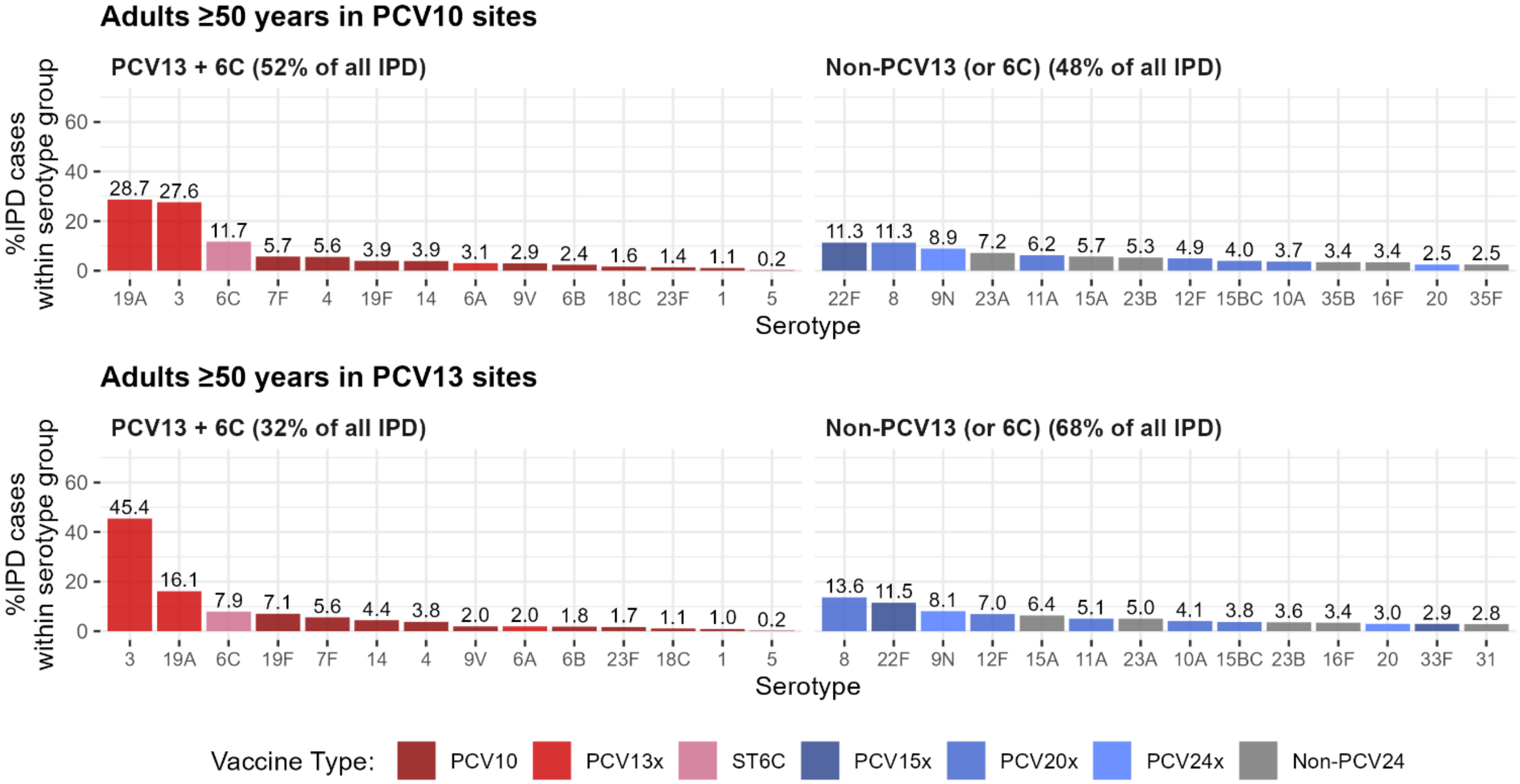

Figure S2. Serotype-specific distribution of IPD in the mature PCV10/13 period, as shown in Figure 3, but with serotypes colored by inclusion in PCV21 or PCV25.

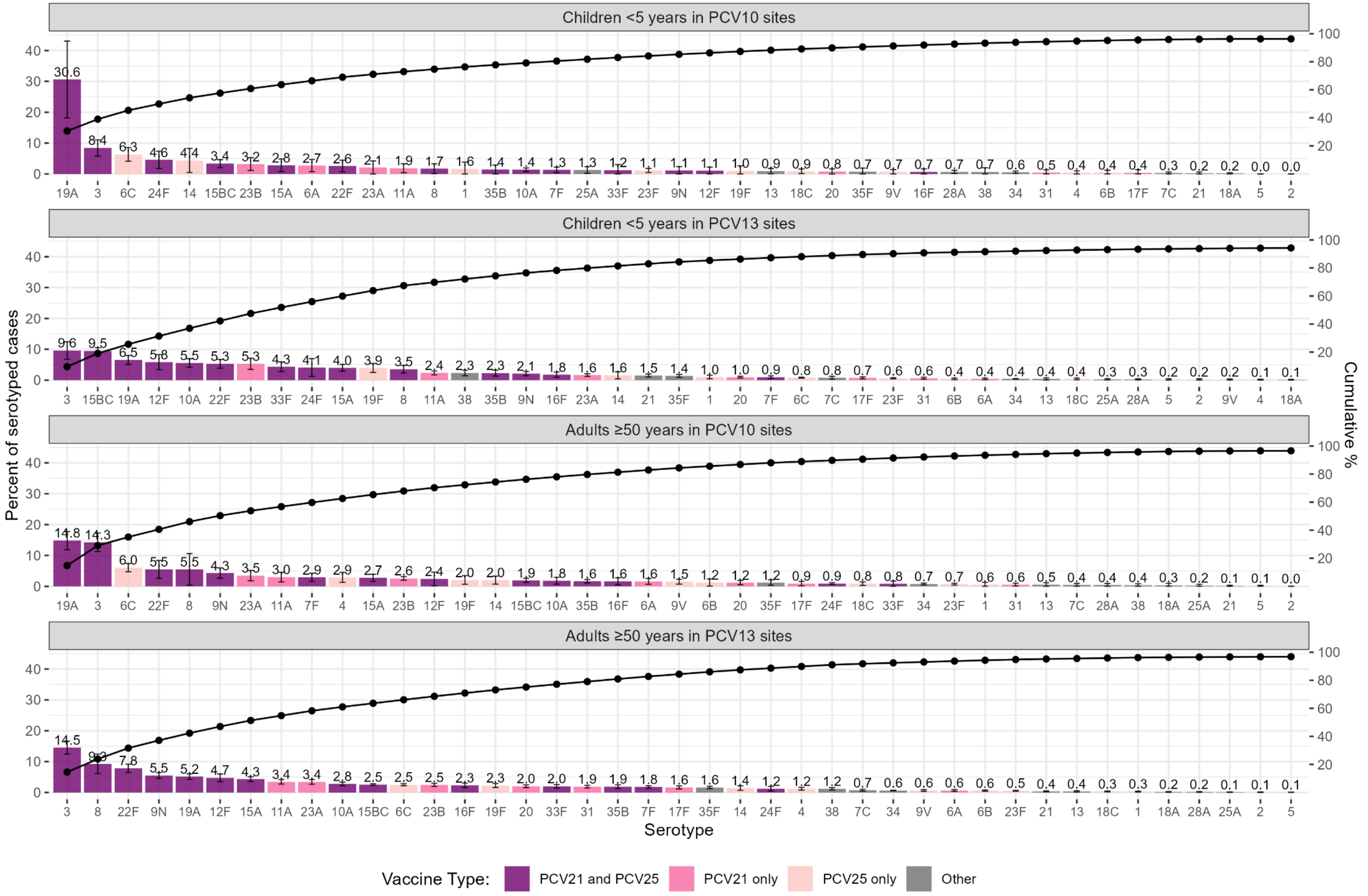

PCV21 and PCV25 = Serotypes included in both PCV21 and PCV25; PCV21 only = Serotypes included in PCV21 but not PCV25; PCV25 only = Serotypes included in PCV25 but not PCV21; Other = Serotypes not included in PCV25 or PCV21.

Figure S3 (A-B). Age distribution of IPD cases at PCV13 sites included in age-stratified analyses, ordered by number of serotyped cases included.

A) Children <5 years

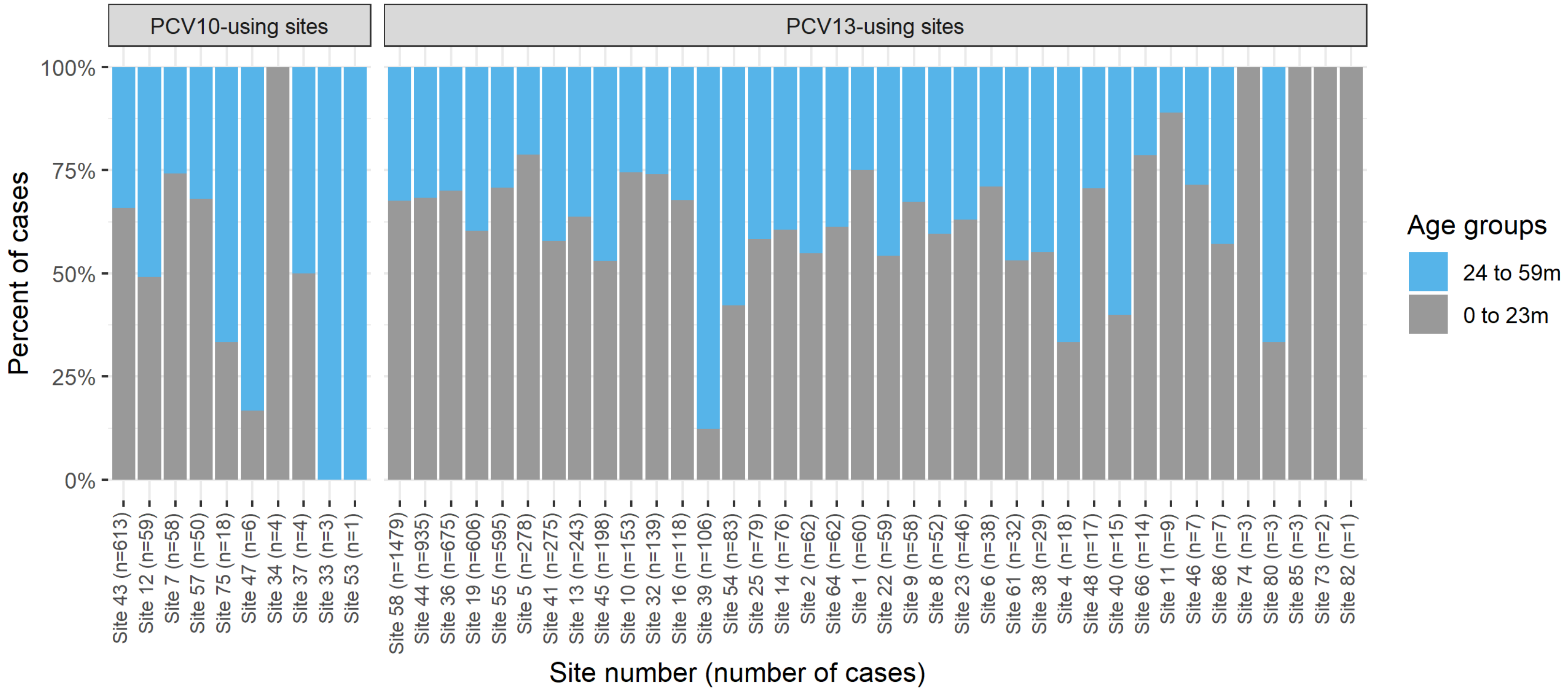

B) Adults ≥50 years in PCV13 sites

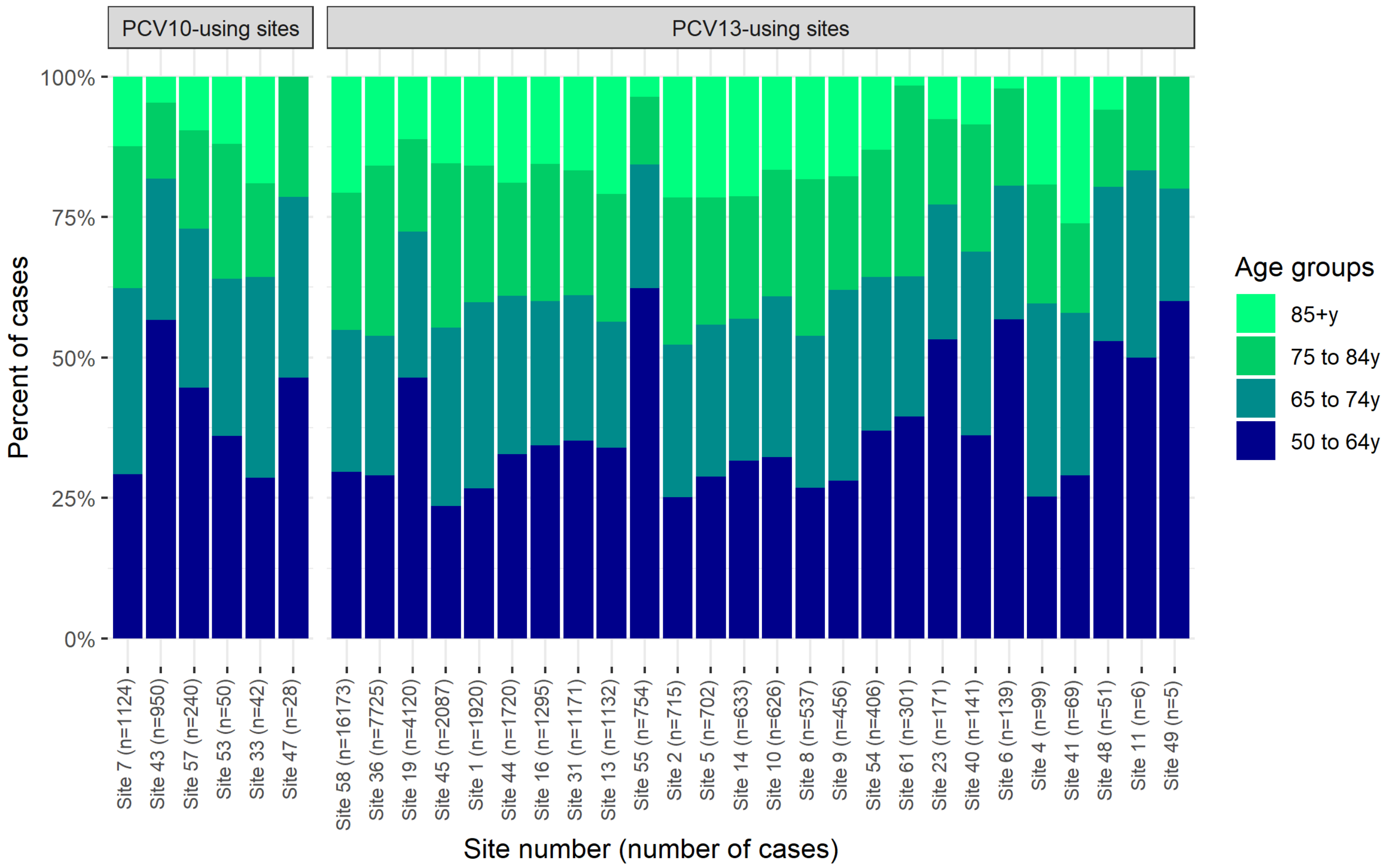

Figure S4 (A-B). Serotype-specific distribution of IPD in the mature PCV13 period, stratified by finer age groups.

A) Children <5 years in PCV13 sites

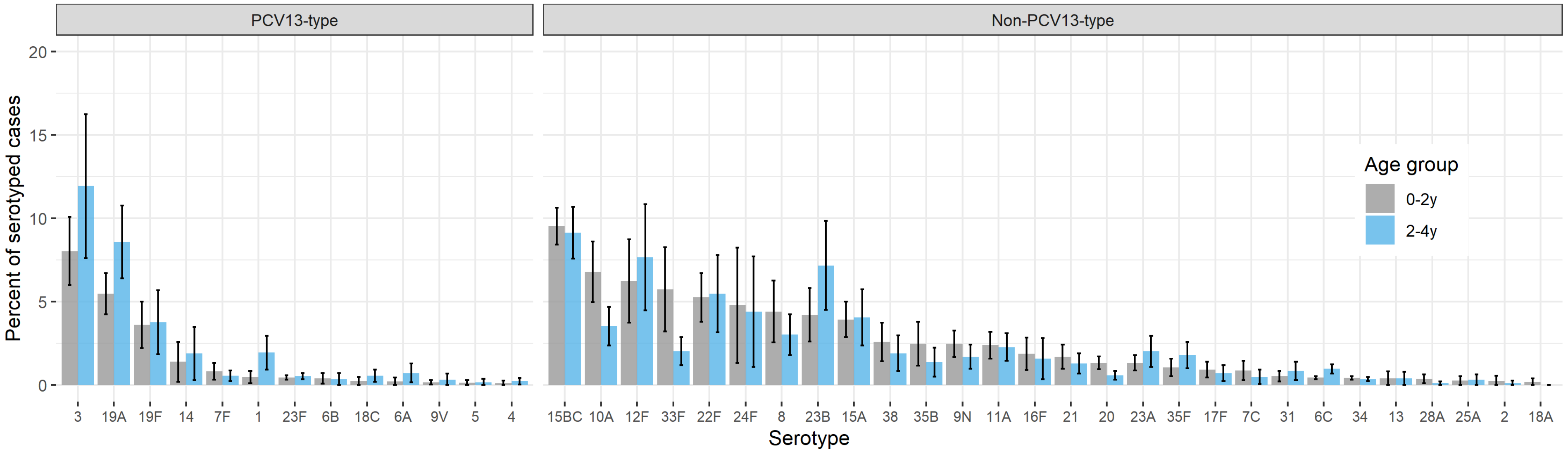

B) Adults ≥50 years in PCV13 sites

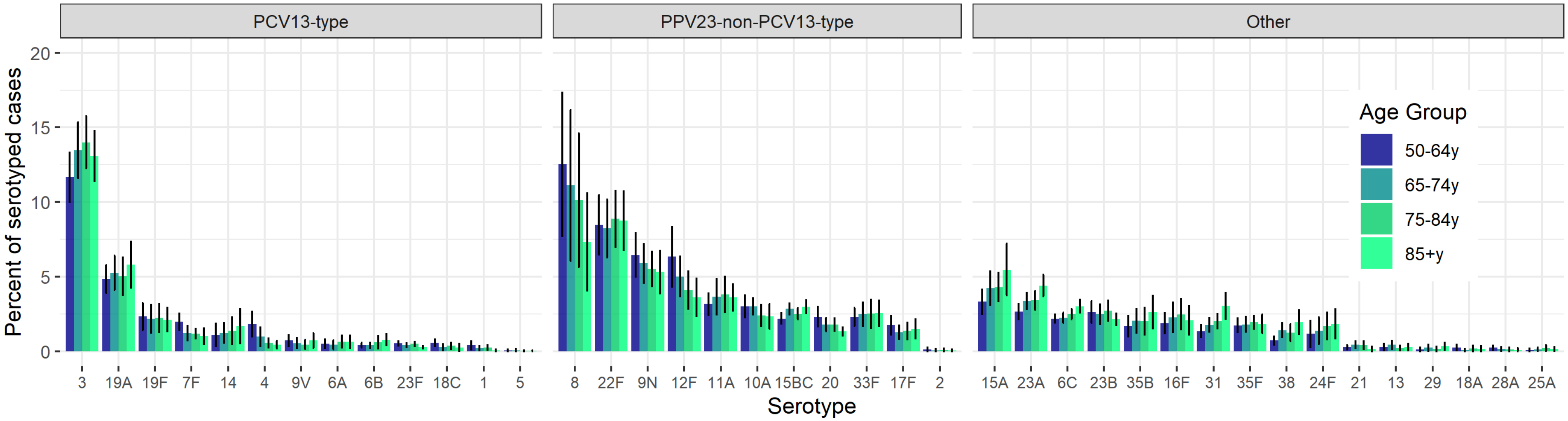

Figure S5 (A-D). Site-specific rank and proportion of IPD due to top 6 serotypes from global meta-average (Figure 2/Table S4) for each age and product strata, colored by region. Blue dashed line and shading represent global result and confidence interval for each serotype. Size of the dot is proportional to the number of cases from each site. Children <5 years are shown in panel A for PCV10 sites and B for PCV13 sites; adults ≥50 years are shown in panel C for PCV10 sites and D for PCV13 sites.

A. Children <5 years in PCV10 sites

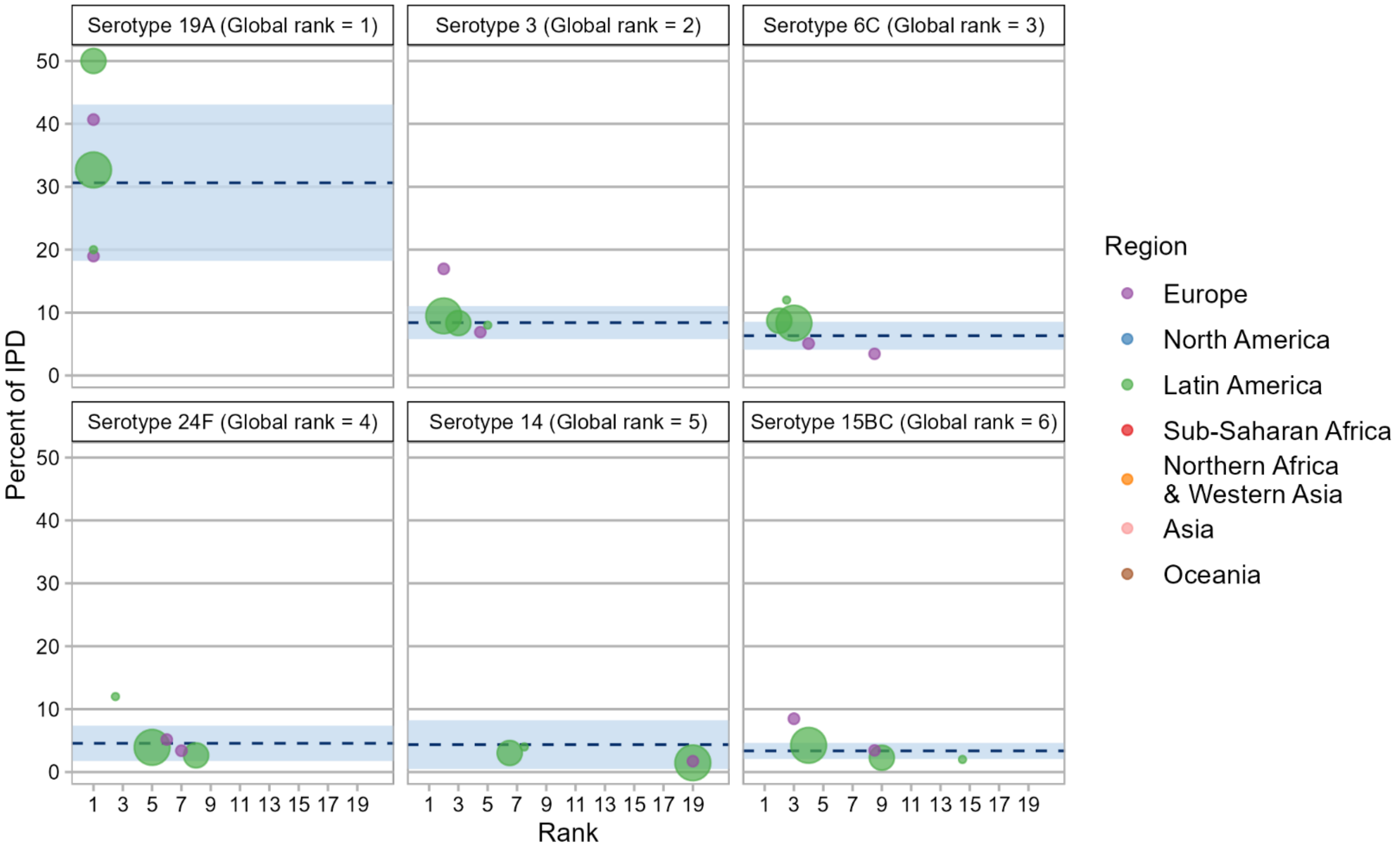

B. Children <5 years in PCV13 sites

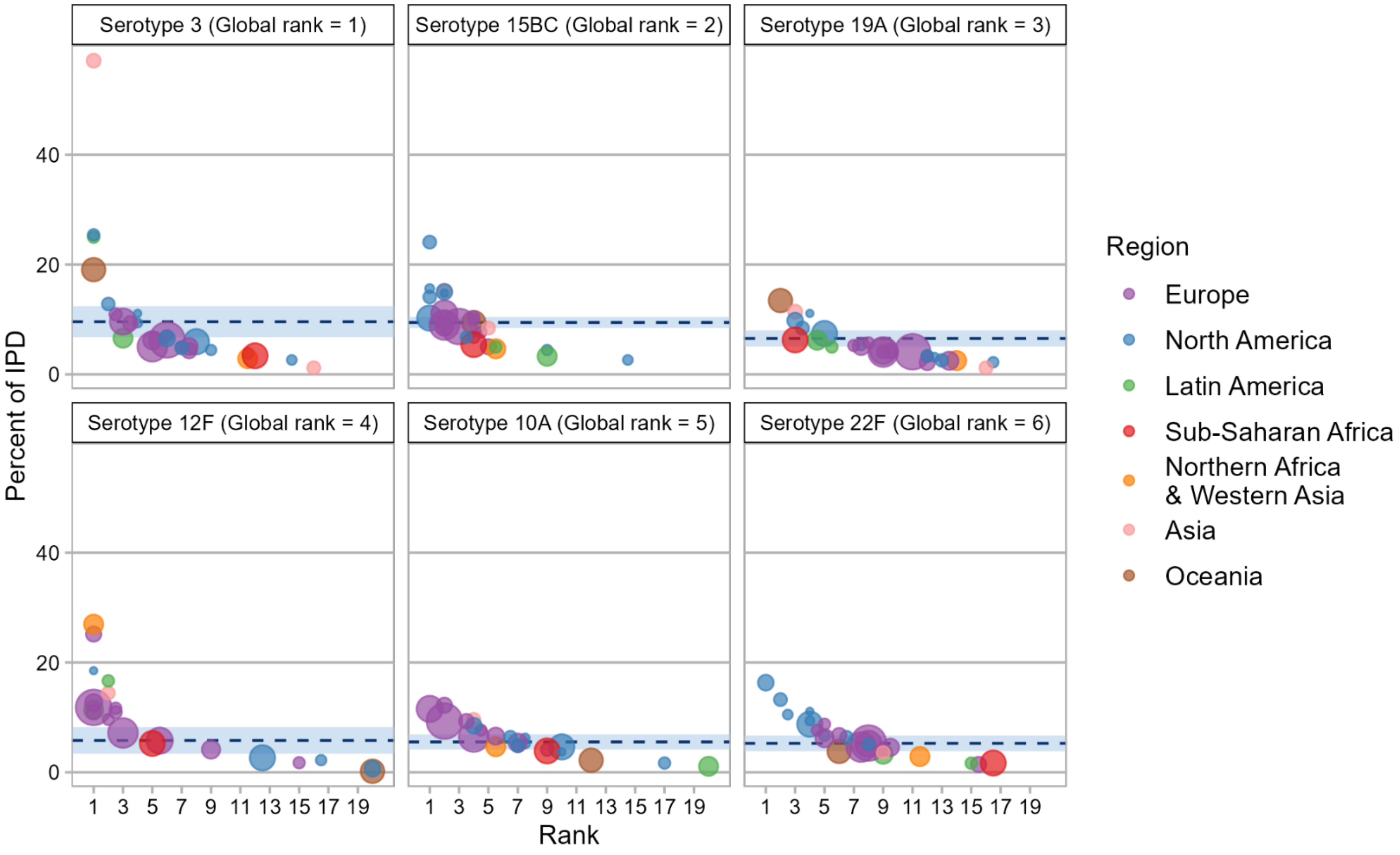

C. Adults ≥50 years in PCV10 sites

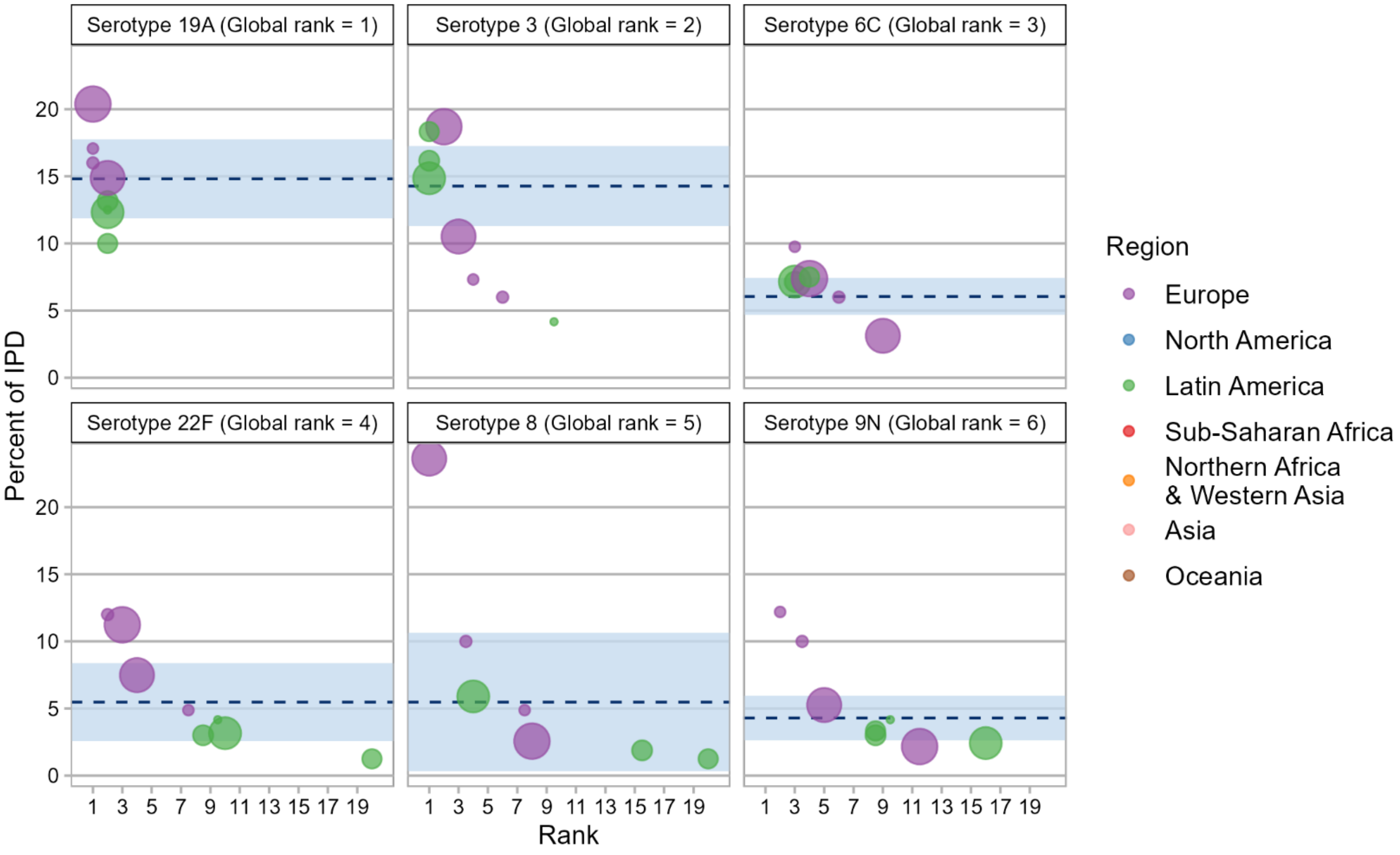

D. Adults ≥50 years in PCV13 sites

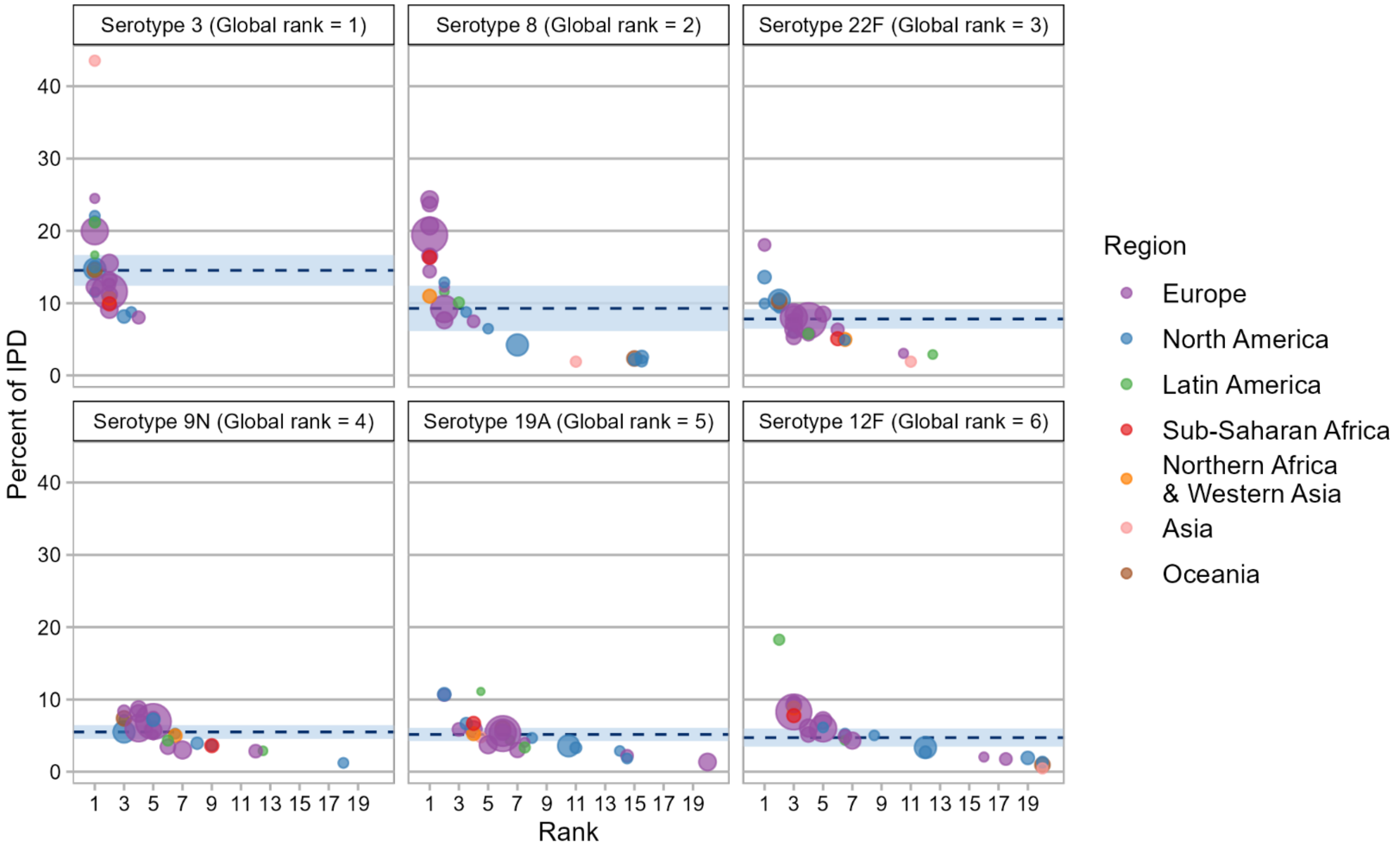

Supplement: Supplement 2 [file NIHMS2058691-supplement-Supplement_2.pdf]
